# Synergistic effect of non-neutralizing antibodies and interferon-γ for cross-protection against influenza

**DOI:** 10.1016/j.isci.2021.103131

**Published:** 2021-09-15

**Authors:** Meito Shibuya, Shigeyuki Tamiya, Atsushi Kawai, Toshiro Hirai, Mark S. Cragg, Yasuo Yoshioka

**Affiliations:** 1 Laboratory of Nano-design for Innovative Drug Development, Graduate School of Pharmaceutical Sciences, Osaka University, 1-6 Yamadaoka, Suita, Osaka 565-0871, Japan; 2 Vaccine Creation Group, BIKEN Innovative Vaccine Research Alliance Laboratories, Institute for Open and Transdisciplinary Research Initiatives, Osaka University, 3-1 Yamadaoka, Suita, Osaka 565-0871, Japan; 3 Vaccine Creation Group, BIKEN Innovative Vaccine Research Alliance Laboratories, Research Institute for Microbial Diseases, Osaka University, 3-1 Yamadaoka, Suita, Osaka 565-0871, Japan; 4 Antibody and Vaccine Group, School of Cancer Sciences, Faculty of Medicine, General Hospital, University of Southampton, Southampton SO16 6YD, UK; 5 The Research Foundation for Microbial Diseases of Osaka University, 3-1 Yamadaoka, Suita, Osaka 565-0871, Japan; 6 Global Center for Medical Engineering and Informatics, Osaka University, 3-1 Yamadaoka, Suita, Osaka 565-0871, Japan

**Keywords:** Biological sciences, Immunology, Immune response

## Abstract

Current influenza vaccines do not typically confer cross-protection against antigenically mismatched strains. To develop vaccines conferring broader cross-protection, recent evidence indicates the crucial role of both cross-reactive antibodies and viral-specific CD4^+^ T cells; however, the precise mechanism of cross-protection is unclear. Furthermore, adjuvants that can efficiently induce cross-protective CD4^+^ T cells have not been identified. Here we show that CpG oligodeoxynucleotides combined with aluminum salts work as adjuvants for influenza vaccine and confer strong cross-protection in mice. Both cross-reactive antibodies and viral-specific CD4^+^ T cells contributed to cross-protection synergistically, with each individually ineffective. Furthermore, we found that downregulated expression of Fcγ receptor IIb on alveolar macrophages due to IFN-γ secreted by viral-specific CD4^+^ T cells improves the activity of cross-reactive antibodies. Our findings inform the development of optimal adjuvants for vaccines and how influenza vaccines confer broader cross-protection.

## Highlights

• The combined use of CpG–ODN and alum improves the efficacy of an influenza vaccine
• Antibodies and CD4^+^ T cells synergistically contribute to cross-protection
• IFN-γ from virus-specific CD4^+^ T cells could confer cross-protection
• The downregulation of FcγRIIb by IFN-γ is associated with cross-protection by antibodies

## Graphical abstract

**Figure:**
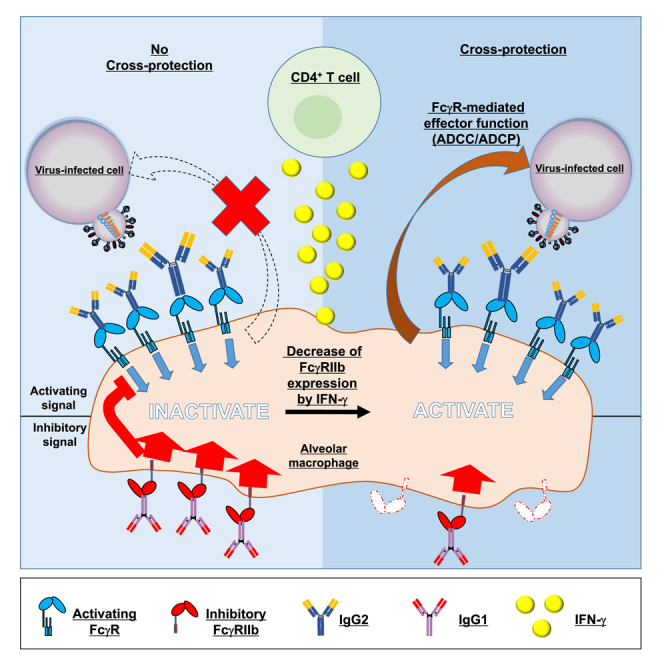

## Introduction

Some of the most prevalent infectious diseases are caused by influenza viruses, contributing to high morbidity and mortality rates worldwide (Krammer et al., 2018). Vaccines can effectively reduce the severity of clinical symptoms and limit the spread of infection (Wei et al., 2020), and several types of vaccines such as split vaccines (SVs) and inactivated whole-virion vaccines (WVs) have been developed for treating influenza virus infections (Wei et al., 2020). Most current influenza vaccines elicit neutralizing antibodies specific for the receptor-binding sites of viral surface hemagglutinin (HA), which are located on the globular head region of HA, to prevent the virus from infiltrating host cells, because this head region exhibits high antigenicity and is important for initiating host cell entry of viruses (Bouvier and Palese, 2008). However, this head region can mutate easily to escape circulating neutralizing antibodies, resulting in constant change of the antigenic properties of HA in seasonal circulating strains—a process known as antigenic drift (Krammer et al., 2018; Wei et al., 2020). Furthermore, the effectiveness of current seasonal influenza vaccines against antigenically mismatched heterologous strains is limited because of this same antigenic drift (Osterholm et al., 2012; Tricco et al., 2013; Zimmerman et al., 2016). This mismatch between vaccine strains and those causing disease will often occur and cannot be avoided despite efforts to predict circulating viruses, thus indicating the need for vaccines with broader cross-protection against heterologous strains.

Apart from developing neutralizing antibodies, several studies have recently shed light on the use of cross-reactive non-neutralizing antibodies for conferring cross-protection against divergent heterologous influenza strains (Krammer and Palese, 2015; Schepens et al., 2018; Wei et al., 2020). Compared to neutralizing antibodies, these cross-reactive non-neutralizing antibodies recognize highly conserved epitopes among influenza viruses, such as the stem region of the HA, but cannot prevent viral entry (Krammer and Palese, 2015; Schepens et al., 2018; Wei et al., 2020). These antibodies can bind to viral proteins present on the surface of infected cells and confer cross-protection via Fcγ receptor (FcγR)-mediated, antibody-dependent cellular cytotoxicity (ADCC) or antibody-dependent cellular phagocytosis (ADCP) (DiLillo et al., 2014, 2016; Krammer and Palese, 2015; Schepens et al., 2018; Wei et al., 2020). In mice, activating FcγRs such as FcγRI, FcγRIII, and FcγRIV contribute to ADCC- or ADCP-mediated cross-protection (Bournazos and Ravetch, 2015; Jegaskanda et al., 2017). The potency of FcγR-mediated effector function is dependent on IgG isotypes (Beers et al., 2016). For example, cross-reactive non-neutralizing mouse IgG2 (mIgG2) is known to provide better cross-protection than mIgG1 in mice, because the binding affinity of mIgG2 for activating FcγRs is stronger than that of mIgG1 (Huber et al., 2006; Van den Hoecke et al., 2017; Vidarsson et al., 2014; Watanabe et al., 2019). In contrast, the sole inhibitory FcγR, FcγRIIb, suppresses the activating FcγRs on effector cells (Nimmerjahn and Ravetch, 2008; Stopforth et al., 2016). The impact of FcγRIIb during the effector phase of infectious disease vaccination, including those for influenza viruses, remains unclear, although the FcγRIIb-mediated suppression is deleterious for direct-targeting antibody therapeutics against cancer (Roghanian et al., 2018). Thus, a better understanding of mIgG1-and mIgG2-mediated FcγRIIb signaling is needed to develop alternative vaccines against influenza viruses that are capable of cross-protection.

In addition to antibodies, CD4^+^ T cells and CD8^+^ T cells induced by influenza virus infection play crucial roles as effector cells in conferring cross-protection (Jansen et al., 2019; Sridhar, 2016). Many reports have shown that viral-specific cytotoxic CD8^+^ T cells, induced by not only virus infection but also vaccines, provide cross-protection, although a high dose of antigen and optimal adjuvants are apparently needed to elicit viral-specific cytotoxic CD8^+^ T cells in influenza vaccines (Budimir et al., 2012; Furuya et al., 2010; Guo et al., 2011; Laidlaw et al., 2013). In contrast, the role of CD4^+^ T cells as effector cells has become an increasing focus of attention recently. Previous reports have shown that viral-specific CD4^+^ T cells induced by prior infection provide cross-protection as effector cells in specific conditions through multiple mechanisms, including help for B cells and CD8^+^ T cells, in addition to direct cytotoxic activity (Devarajan et al., 2016, 2018; Juno et al., 2017; McKinstry et al., 2012). However, compared to CD8^+^ T cells, the role of viral-specific CD4^+^ T cells as effector cells remains unclear and is still debated (Devarajan et al., 2016, 2018). Considering the potential of cross-reactive non-neutralizing antibodies, it is essential to elucidate not only the precise contribution of viral-specific CD4^+^ T cells as effector cells for cross-protection but also the potentially synergistic effects of CD4^+^ T cells and cross-reactive non-neutralizing antibodies.

One means for improving the efficacy of influenza vaccines capable of conferring cross-protection is the use of appropriate adjuvants. We recently demonstrated the importance of the use of rational adjuvants in providing cross-protective activity in an influenza vaccine by using oligodeoxynucleotides (ODN) with unmethylated cytosine-phosphate-guanine (CpG) motifs (CpG–ODN), which are toll-like receptor 9 (TLR9) agonists (Shirota and Klinman, 2014) and aluminum salts (alum), which are commonly used globally (HogenEsch et al., 2018). We showed that CpG–ODN-adjuvanted influenza vaccine, which induces predominantly viral-specific mIgG2b and mIgG2c, confers stronger cross-protection against heterologous virus challenge via ADCC or ADCP than alum-adjuvanted vaccine, which predominantly induces viral-specific mIgG1 (Shibuya et al., 2020). Furthermore, we demonstrated a new concept that vaccine-induced cross-reactive non-neutralizing mIgG1 anti-viral antibodies suppressed the cross-protective effects of mIgG2b/c against a heterologous virus challenge (Shibuya et al., 2020). These results suggest that an adjuvant that induces viral-specific mIgG2b/c selectively, such as CpG–ODN, is optimal for heterologous protection. Furthermore, because each adjuvant induces unique antibody and T cell responses (Ciabattini et al., 2016; Martins et al., 2016; McKee and Marrack, 2017), this study suggested that the evaluation of vaccine-induced cross-protection using several types of adjuvants would allow elucidation of the relationship between cross-reactive non-neutralizing antibodies, FcγR-mediated cross-protection, and T cells.

In this study, we demonstrate the ability of the combination of CpG–ODN and alum (CpG/alum) to confer strong cross-protection in mice. Furthermore, we show that downregulation of FcγRIIb on alveolar macrophages by IFN-γ from viral-specific CD4^+^cells improves the activity of cross-reactive non-neutralizing antibodies. Thus, our findings could help in selecting optimal adjuvants for vaccines and developing influenza vaccines with broader cross-protection.

## Results

### Antibody and T cell responses are optimally induced by the combination of CpG–ODN and alum

**Figure 1:**
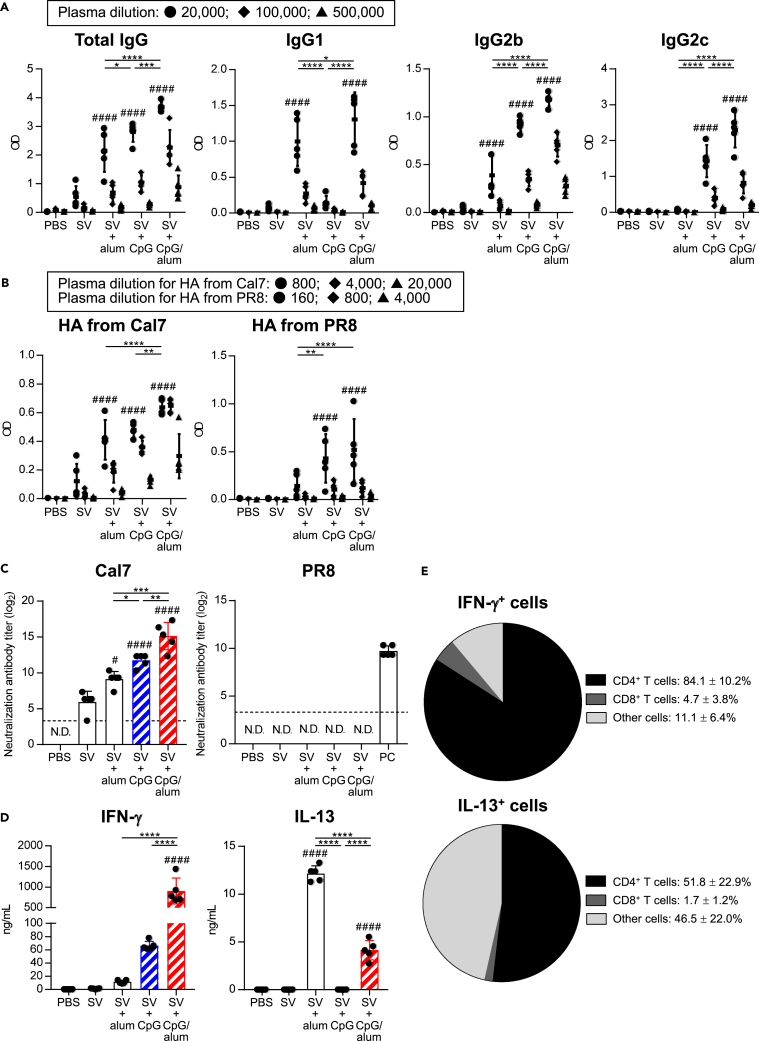
Antibody responses after the combined use of CpG–ODN and alum as adjuvants Mice were immunized with SV alone, SV plus alum, SV plus CpG–ODN, or SV plus CpG/alum subcutaneously. As a control, mice were treated with PBS subcutaneously. (A and B) Levels of (A) SV-specific total mIgG, mIgG1, mIgG2b, and mIgG2c in the plasma, and (B) rHA from Cal7-and rHA from PR8-specific total mIgG in the plasma were determined after final immunization. We used (A) 20,000- (●), 100,000- (◆), and 500,000- (▲) fold–diluted plasma samples,(B) 800- (●), 4,000- (◆), and 20,000- (▲) fold–diluted plasma samples for rHA from Cal7, and 160- (●), 800- (◆), and 4,000- (▲) fold-diluted plasma samples for rHA from PR8. (C) Neutralization titers against Cal7 and PR8 in the plasma samples from immunized mice were determined using MDCK cells. Plasma samples from PR8-immunized mice were used as a positive control (PC). N.D.: not detected. The dashed line shows the detection threshold for a positive response. (D) Splenocytes obtained from immunized mice were incubated in the presence of SV *in vitro* and the levels of IFN-γ and IL-13 in the supernatants were measured after 3 days. (E) Splenocytes obtained from SV plus CpG/alum-immunized mice were incubated in the presence of SV with the protein transport inhibitor cocktail for 24 h *in vitro* and the intracellular IFN-γ and IL-13 levels were evaluated by flow cytometry.(A–E) *n* = 5 per group. Data are means ± SD. ^#^p<0.05, ^####^p<0.0001 vs. SV alone group; ∗p<0.05, ∗∗p<0.01, ∗∗∗p<0.001, ∗∗∗∗p<0.0001 as indicated by Tukey’s test. Significant differences were analyzed only in the (A) 20,000-fold-diluted plasma samples, (B) 800-fold-diluted plasma samples for rHA from Cal7 and 160-fold-diluted plasma samples for rHA from PR8. See also Figures S1 and S2.

First, we examined the characteristics of the antibody responses induced by the combined use of CpG–ODN and alum (CpG/alum) as adjuvants in mice. Conventional SV from the H1N1 influenza A virus (strain: A/California/7/2009 (Cal7)) was used as antigen. Mice were immunized with SV alone, SV plus alum, SV plus CpG–ODN, or SV plus CpG/alum and the levels of SV-specific total mIgG, mIgG1, mIgG2b, and mIgG2c were measured in the plasma after the last immunization (Figure 1A). As reported previously (Shibuya et al., 2020), SV plus CpG–ODN induced significantly higher levels of SV-specific mIgG2b and mIgG2c than SV alone and SV plus alum, whereas the level of SV-specific mIgG1 in SV plus alum-immunized mice was significantly higher than that in SV alone-immunized mice and SV plus CpG–ODN-immunized mice (Figure 1A). Mice immunized with SV plus CpG/alum showed significantly higher levels of SV-specific total mIgG, mIgG1, mIgG2b, and mIgG2c than those immunized with SV plus alum and SV plus CpG–ODN (Figure 1A).

To compare the cross-protective activity, we examined the levels of Cal7-derived recombinant HA- and H1N1 A/Puerto Rico/8/34 (PR8)-derived recombinant HA-specific total mIgG in the plasma of immunized mice (Figure 1B). PR8 is a heterologous strain obtained from the one used as the vaccine antigen. Consistent with the levels of SV-specific total mIgG, the levels of total mIgG specific for HA from Cal7 in SV plus CpG/alum-immunized mice were significantly higher than those in SV plus alum-immunized mice and SV plus CpG–ODN-immunized mice (Figure 1B). In addition, SV plus CpG/alum and SV plus CpG–ODN induced significantly higher levels of total mIgG specific for HA from PR8 than SV alone, although the level of total mIgG specific for HA from PR8 was lower than that of HA from Cal7 (Figure 1B). Next, we examined the neutralizing activity (Figure 1C) and hemagglutination inhibition (HI) titers (Figure S1) against homologous Cal7 and heterologous PR8 by using plasma samples obtained from immunized mice. The neutralizing activities (Figure 1C) and HI titers (Figure S1) for Cal7 showed the same trend as the levels of SV-specific total mIgG, and mice immunized with SV plus CpG/alum had a significantly higher neutralizing activity and HI titer than those immunized with SV plus alum and SV plus CpG–ODN. Furthermore, we did not observe any neutralizing activities and HI titers for PR8 in any of the plasma samples except the positive control (Figures 1C and S1). Collectively, these results suggested that the combined use of CpG–ODN and alum enhance neutralizing antibody responses against a homologous virus and cross-reactive non-neutralizing antibody responses against a heterologous virus compared with the individual use of the adjuvants.

To investigate SV-specific CD4^+^T cell responses, splenocytes from immunized mice were stimulated with SV *in vitro*, and we measured the levels of IFN-γ and IL-13 in the supernatant (Figure 1D). The IFN-γ level in SV plus CpG–ODN-immunized mice was higher than that in SV alone- and SV plus alum-immunized mice, while the IL-13 level in SV plus alum-immunized mice was significantly higher than that in SV alone- and SV plus CpG–ODN-immunized mice (Figure 1D). Mice immunized with SV plus CpG/alum showed significantly higher levels of IFN-γ than those immunized with SV plus alum and SV plus CpG–ODN, although the IL-13 level in SV plus CpG/alum-immunized mice was significantly lower than that in SV plus alum-immunized mice (Figure 1D). In addition, we examined the cellular origin of the IFN-γ in SV plus CpG/alum-immunized mice by intracellularly staining for IFN-γ after stimulation with SV *in vitro* (Figures 1E and S2). We found that CD4^+^ T cells represented >80% of IFN-γ-producing splenocytes and roughly 50% of IL-13 producing splenocytes after stimulation with SV (Figures 1E and S2). These results indicate that the combined use of CpG–ODN and alum enhances the Th1 response compared with the use of CpG–ODN alone with a corresponding decrease in the Th2 response compared with the use of alum alone. They also demonstrate that CD4^+^ T cells represent the major source of IFN-γ after such immunization.

### SV plus CpG/alum confers strong cross-protection against heterologous virus challenge

**Figure 2:**
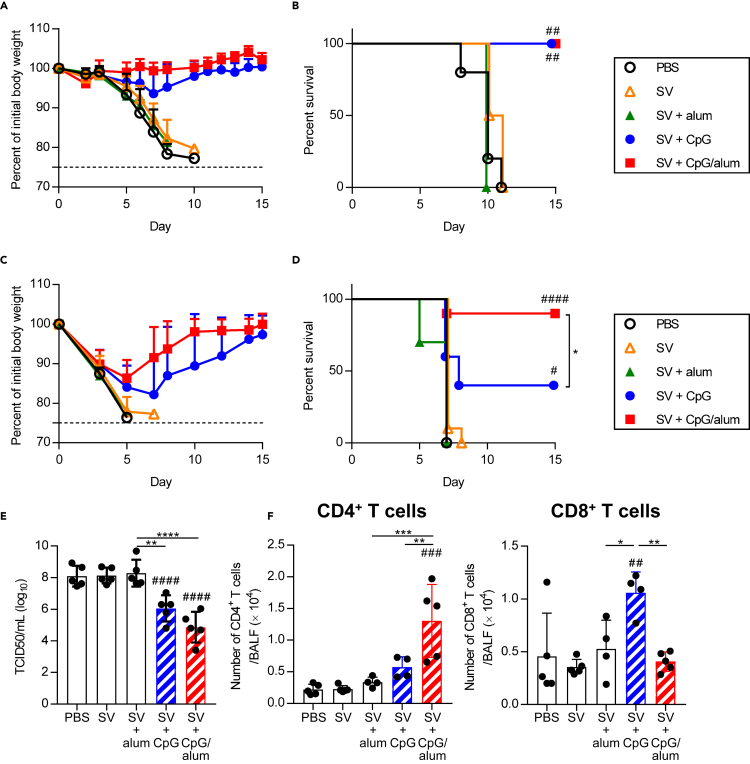
Cross-protection against heterologous PR8 challenge Mice were immunized as in Figure 1. Ten days after the final immunization, mice were challenged with PR8. (A–F) After challenge with (A and B) 1.2 × 10 TCID_50_ PR8 and (C–F) 1.2 × 10^3^ TCID_50_ PR8, we monitored (A and C) percentages of initial body weights and (B and D) survival for the next 15 days. Five days after challenge with 1.2 × 10^3^ TCID_50_ PR8, (E) virus titers and (F) the numbers of CD4^+^ T cells and CD8^+^ T cells in BALF were measured. (A and B) *n* = 4–5, (C and D) *n* = 10, (E) *n* = 5, (F) *n* = 4–5 per group. (A, C, E and F) Data are means ± SD. (B and D)^#^p<0.05, ^##^p<0.01, ^####^p<0.0001 vs. SV alone group; ∗p<0.05 as indicated by comparing Kaplan–Meier curves using the log-rank test.(E and F)^##^p<0.01, ^###^p<0.001, ^####^p<0.0001 vs. SV alone group; ∗p<0.05, ∗∗p<0.01, ∗∗∗p<0.001, ∗∗∗∗p<0.0001 as indicated by Tukey’s test.

After immunization, the mice were challenged with heterologous PR8 and we observed the changes in body weight (Figures 2A and 2C) and survival (Figures 2B and 2D). After a challenge with a low virus titer, all the mice in the PBS-treated control group, SV alone-immunized mouse group, and SV plus alum-immunized mouse group died within 11 days (Figures 2A and 2B). In contrast, all SV plus CpG–ODN-immunized mice and SV plus CpG/alum-immunized mice survived without any body weight loss (Figures 2A and 2B). In addition, after a challenge with a high virus titer, only 40% of SV plus CpG–ODN-immunized mice survived, while all the mice in the PBS-treated control group, SV alone-immunized mouse group, and SV plus alum-immunized mouse group died within 10 days (Figures 2C and 2D). 90% of SV plus CpG/alum-immunized mice survived, and their body weights recovered after an initial loss (Figures 2C and 2D). The virus titers in the bronchoalveolar lavage fluid (BALF) of SV plus CpG/alum-immunized mice and SV plus CpG–ODN-immunized mice were significantly lower than those in the PBS-treated control group on day 5 after the challenge, whereas a lower but not significantly different virus titer was observed in the SV plus CpG/alum-immunized mice compared with that in the SV plus CpG–ODN-immunized mice (Figure 2E). In addition, the number of CD4^+^ T cells in BALF was significantly higher in the group immunized with SV plus CpG/alum than in the groups immunized with SV alone, SV plus alum, and SV plus CpG–ODN (Figure 2F). In contrast, the number of CD8^+^ T cells in the BALF was significantly higher in mice immunized with SV plus CpG–ODN than in SV alone-immunized mice, SV plus alum-immunized mice, and SV plus CpG/alum-immunized mice (Figure 2F). These data suggest that SV plus CpG/alum provides superior cross-protection against a heterologous virus challenge compared with SV plus CpG–ODN, although it does not induce neutralizing antibodies against the heterologous virus.

### Both cross-reactive non-neutralizing antibodies and CD4^+^ T cells contribute to SV plus CpG/alum-induced cross-protection

**Figure 3:**
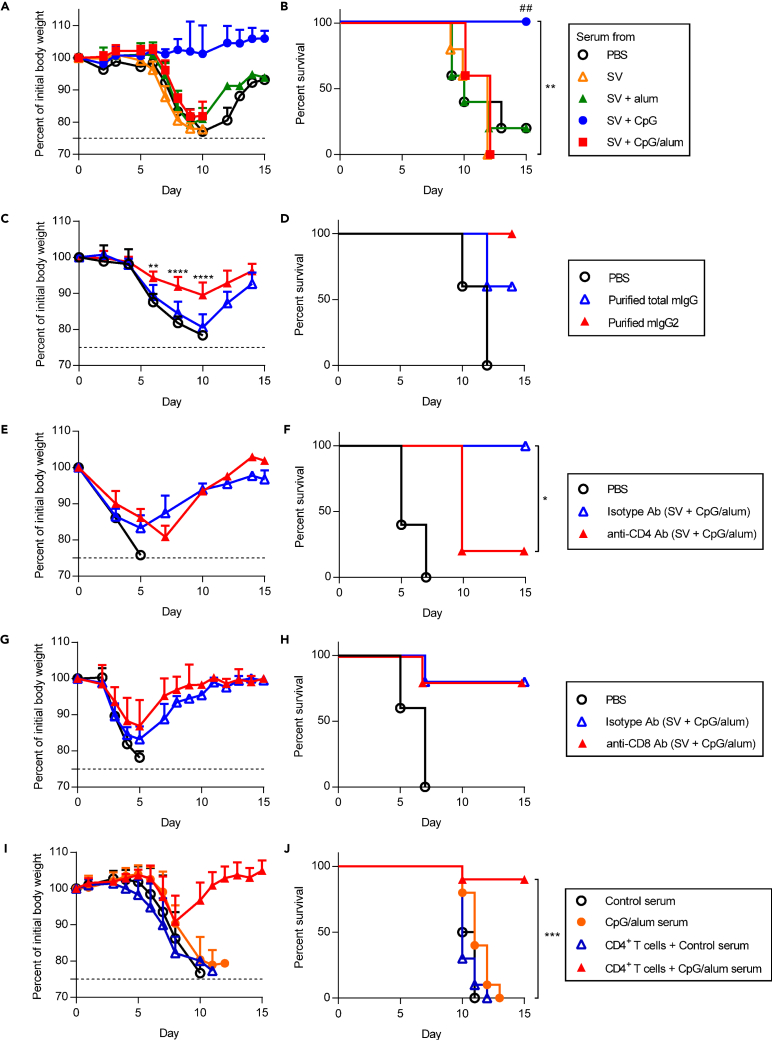
Synergistic effects of cross-reactive non-neutralizing antibodies and CD4^+^ T cells for cross-protection (A and B) A mixture of 1.2 × 10 TCID_50_ PR8 and 2 fold diluted serum from PBS-treated control mice, SV plus alum-, SV plus CpG–ODN-, or SV plus CpG/alum-immunized mice was transferred into naive mice intranasally. We monitored (A) percentages of initial body weights and (B) survival for the next 15 days. (C and D) A mixture of 6 TCID_50_ PR8 and purified total mIgG or mIgG2 from the serum of SV plus CpG/alum-immunized mice was transferred into naive mice intranasally. Percentages (C) of initial body weights and (D) survival were monitored for the next 15 days. (E–H) After immunization with SV plus CpG/alum, (E and F) anti-CD4 antibody or isotype antibody, and (G and H) anti-CD8 antibody or isotype antibody was injected into mice before and during 1.2 × 10^3^ TCID_50_ PR8 challenge. As control, mice treated with PBS were challenged with 1.2 × 10^3^ TCID_50_ PR8. We monitored (E and G) percentages of initial body weights and (F and H) survival for the next 15 days. (I and J) CD4^+^ T cells from splenocytes from SV plus CpG/alum-immunized mice were injected into naive recipient mice intravenously. Mixture of 1.2 × 10 TCID_50_ PR8 and 2 fold diluted serum from PBS-treated control mice or SV plus CpG/alum-immunized mice was transferred intranasally into naive mice or CD4^+^ T cells-transferred mice. We monitored (I) percentages of initial body weights and (J) survival for the next 15 days. (A–H) *n* = 5, (I, J) *n* = 10 per group. (A, C, E, G and I) Data are means ± SD. (B)^##^p<0.01 vs. SV alone group; ∗∗p<0.01 between serum from SV plus CpG–ODN group vs. serum from SV plus CpG/alum group; (F) ∗p<0.05; (J) ∗∗∗p<0.001 between CD4^+^ T cells plus serum from SV plus CpG/alum group vs. serum from SV plus CpG/alum group as indicated by comparing Kaplan–Meier curves using the logrank test.(C) ∗∗p<0.01, ∗∗∗∗p<0.0001 between purified total mIgG group vs. purified mIgG2 group as indicated by Bonferroni’s test (excluding data after day 10 post infection from analysis when mice in the purified total mIgG group were culled due to reaching terminal endpoints). See also Figures S3 and S4.

To examine the contribution of cross-reactive non-neutralizing antibodies for conferring a strong cross-protection by the CpG/alum-adjuvanted vaccine, serum samples from immunized or PBS-treated control mice were mixed with PR8 *in vitro* and transferred into naive mice intranasally (Figures 3A and 3B (low virus titer) and Figure S3 (high virus titer)). As reported previously (Shibuya et al., 2020), the serum obtained from mice immunized with SV plus CpG–ODN suppressed body weight loss and improved the survival upon transfer than that obtained from PBS-treated control mice, while the serum obtained from mice immunized with SV plus alum did not improve body weight loss and survival (Figures 3A, 3B and S3). Furthermore, the serum obtained from mice immunized with SV plus CpG/alum did not show an improvement in body weight loss and survival upon transfer (Figures 3A, 3B and S3). These data indicate that cross-reactive non-neutralizing antibodies alone induced by SV plus CpG/alum do not provide cross-protection against a heterologous virus challenge.

Next, we examined the cross-protective activity of mIgG2 purified from the serum of mice immunized with SV plus CpG/alum and compared it with purified total mIgG. We confirmed that the levels of SV-specific mIgG1 in the purified mIgG2 fraction were lower than those in the same amount of purified total mIgG, whereas there was no difference in the levels of SV-specific mIgG2b and mIgG2c between these groups (Figure S4). Next, purified total mIgG or mIgG2 were mixed with PR8 *in vitro* and transferred into naive mice intranasally (Figures 3C and 3D). Mice that received the mixture of PR8 and purified mIgG2 showed improved body weight loss and survival rates upon transfer compared with those that received the PR8 and purified total mIgG mixture (Figures 3C and 3D). These data suggest that the cross-reactive non-neutralizing mIgG1 present in the total mIgG from immunized mice inhibit the cross-protective activity of cross-reactive non-neutralizing mIgG2.

Next, to examine the involvement of T cells as effector cells for cross-protection against a heterologous virus challenge in SV plus CpG/alum-immunized mice, CD4^+^ or CD8^+^ T cells were depleted in SV plus CpG/alum-immunized mice during the PR8 challenge (Figures 3E–3H). The depletion of CD4^+^ T cells in these mice caused a significantly decreased survival compared with non-depleted immunized mice (Figures 3E and 3F). On the other hand, we did not observe a change in body weight loss or survival after the depletion of CD8^+^ T cells compared with non-depleted immunized mice (Figures 3G and 3H). These results indicate that CD4^+^ T cells play a crucial role in conferring cross-protection against a heterologous virus challenge in SV plus CpG/alum-immunized mice.

Next, we confirmed the contribution of CD4^+^ T cells for cross-protection in CpG/alum-adjuvanted mice (Figures 3I and 3J). We also examined the synergistic effect of cross-reactive non-neutralizing antibodies and CD4^+^ T cells for cross-protection in SV plus CpG/alum-immunized mice (Figures 3I and 3J). CD4^+^ T cells obtained from SV plus CpG/alum-immunized mice were adoptively transferred to naive mice intravenously and these mice were challenged intranasally with a mixture of PR8 and serum from PBS-treated control mice (Figures 3I and 3J). We did not observe an improvement in body weight loss and survival in mice that received CD4^+^ T cells from SV plus CpG/alum-immunized mice (blue triangle in Figures 3I and 3J) compared with mice that received the mixture of PR8 and serum from PBS-treated control mice (black circle in Figures 3I and 3J), indicating that viral-specific CD4^+^ T cells alone are not sufficient to protect against a heterologous PR8 challenge. In contrast, administration of both the serum from SV plus CpG/alum-immunized mice and CD4^+^ T cells from SV plus CpG/alum-immunized mice prevented body weight loss and improved the survival after the PR8 challenge (red triangle in Figures 3I and 3J). These results suggest that both cross-reactive non-neutralizing antibodies and CD4^+^ T cells play a crucial role by conferring cross-protection synergistically.

### Both IFN-γ secreted by CD4^+^ T cells and cross-reactive non-neutralizing antibodies contribute to cross-protection

**Figure 4:**
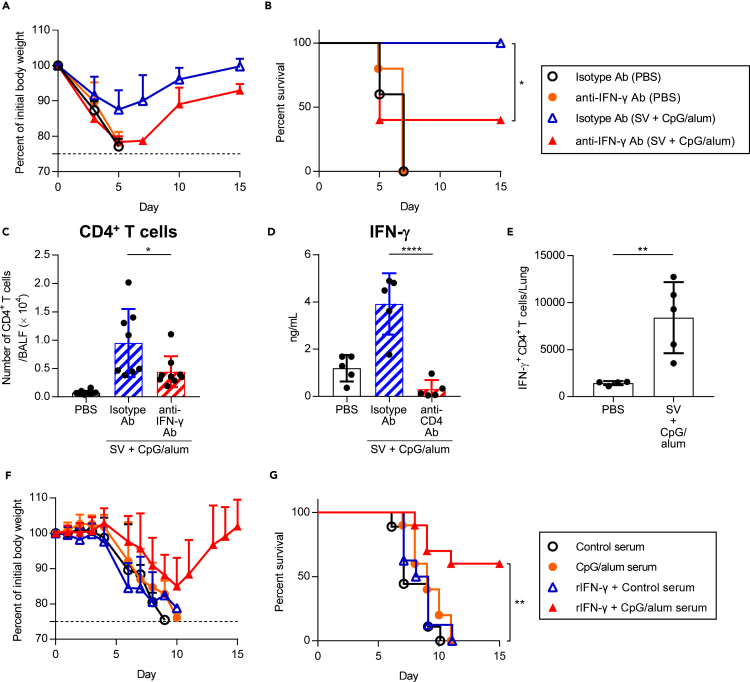
Requirement of IFN-γ for cross-protection (A–C) After treatment with PBS or immunization with SV plus CpG/alum, anti-IFN-γ antibody or isotype antibody was injected into mice before 1.2 × 10^3^ TCID_50_ PR8 challenge. We monitored (A) percentages of initial body weights and (B) survival for the next 15 days. (C) Five days after PR8 challenge, the number of CD4^+^ T cells in BALF was measured. (D) After treatment with PBS or immunization with SV plus CpG/alum, anti-CD4 antibody or isotype antibody was injected into mice before and during the 1.2 × 10^3^ TCID_50_ PR8 challenge. Five days after the PR8 challenge, the level of IFN-γ in BALF was measured. (E) After treatment with PBS or immunization with SV plus CpG/alum, mice were challenged with 1.2 × 10^3^ TCID_50_ PR8. Four days after challenge, mice were treated with Brefeldin A intraperitoneally to block cytokine secretion, followed by harvesting of the lung after 6 h. Single cell suspensions were prepared and intracellular IFN-γ was analyzed by flow cytometry. (F and G) Mixture of 1.2 × 10 TCID_50_ PR8 and 2 fold diluted serum from PBS-treated control mice or SV plus CpG/alum-immunized mice was transferred into naive mice intranasally. These mice were treated with recombinant IFN-γ, and we monitored (F) percentages of initial body weights and (G) survival for the next 15 days. (A, B, D and E) *n* = 5, (C, F and G) *n* = 8–10 per group. (A, C, D, E and F) Data are means ± SD. (B) ∗p<0.05 as indicated by comparing Kaplan–Meier curves using the logrank test. (C and D) ∗p<0.05, ∗∗∗∗p<0.0001 as indicated by Tukey’s test. (E) ∗∗p<0.01 as indicated by Student’s ttest. (G) ∗∗p<0.01 between recombinant IFN-γ plus serum from SV plus CpG/alum group vs. serum from SV plus CpG/alum group as indicated by comparing Kaplan–Meier curves using the logrank test. See also Figures S5 and S6.

We speculated that the cytokines secreted by CD4^+^ T cells contribute to the cross-protection along with cross-reactive non-neutralizing antibodies in SV plus CpG/alum-immunized mice. We focused on IFN-γ, because IFN-γ production from splenocytes in SV plus CpG/alum-immunized mice was highly elevated as shown in Figure 1D. PBS-treated control mice and SV plus CpG/alum-immunized mice were treated with anti-IFN-γ neutralizing antibody during the PR8 challenge (Figures 4A and 4B). PBS-treated control mice treated with anti-IFN-γ antibody showed no significant differences in body weight loss and survival (Figures 4A and 4B). On the other hand, SV plus CpG/alum-immunized mice treated with anti-IFN-γ antibody exhibited severe weight loss and morbidity unlike those exposed to isotype-control antibody, indicating the crucial role of IFN-γ in conferring cross-protection (Figures 4A and 4B). In addition, the number of CD4^+^ T cells in the BALF of SV plus CpG/alum-immunized mice was significantly decreased after treatment with anti-IFN-γ antibody compared with isotype control antibody on day 5 after the challenge (Figure 4C). We also observed that the IFN-γ level in the BALF of SV plus CpG/alum-immunized mice was significantly decreased after the depletion of CD4^+^ T cells during the PR8 challenge compared with non-depleted immunized mice (Figure 4D). In addition, we confirmed, using intracellular staining for IFN-γ, that the presence of CD4^+^ T cells in the lungs produced IFN-γ in SV plus CpG/alum-immunized mice on day 4 after the challenge (Figures 4E and S5). These results suggest that IFN-γ secreted by CD4^+^ T cells plays an important role in conferring cross-protection in SV plus CpG/alum-immunized mice.

To further verify the synergistic action of IFN-γ and cross-reactive non-neutralizing antibodies in conferring cross-protection in SV plus CpG/alum-immunized mice, serum from SV plus CpG/alum-immunized mice was mixed with PR8 *in vitro* and transferred to naive mice intranasally (Figures 4F and 4G). These mice were also treated with recombinant IFN-γ intranasally during the challenge (Figures 4F and 4G). As described in Figure 2A, the serum from SV plus CpG/alum-immunized mice did not improve body weight loss and survival upon PR8 challenge (Figures 4F and 4G). On the other hand, treatment with both recombinant IFN-γ and the serum from SV plus CpG/alum-immunized mice improved body weight loss and survival upon PR8 challenge, although treatment with both recombinant IFN-γ and the serum from PBS-treated control mice did not induce cross-protection (Figures 4F and 4G). In addition, treatment with recombinant IFN-γ improved cross-protection induced by the serum from SV plus alum-immunized mice (Figure S6). These data suggest that both IFN-γ and non-neutralizing antibodies are needed and sufficient for conferring cross-protection in SV plus CpG/alum-immunized mice.

### Decreasing the expression of FcγRIIb on alveolar macrophages by IFN-γ contributes to cross-protection

**Figure 5:**
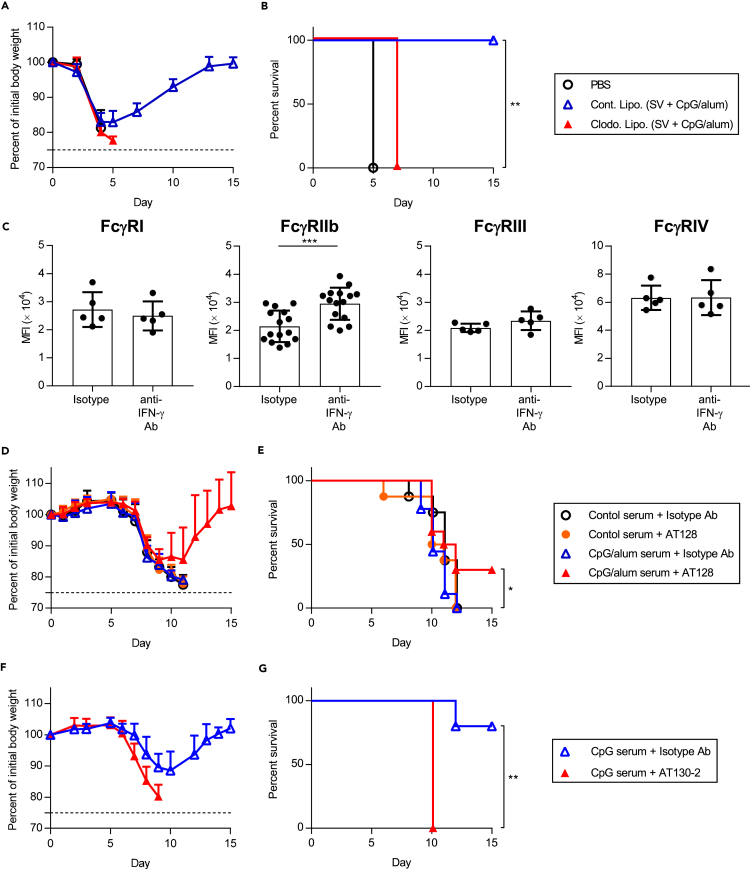
Changes in the expression of FcγRs on alveolar macrophages and the negative role of FcγRIIb in cross-protection (A and B) After immunization with SV plus CpG/alum, clodronate liposomes or control liposomes were administered to mice intranasally. After the 1.2 × 10^3^ TCID_50_ PR8 challenge, we monitored (A) percentages of initial body weights and (B) survival for the next 15 days. Mice treated with PBS were challenged with 1.2 × 10^3^ TCID_50_ PR8 and used as control. (C) After immunization with SV plus CpG/alum, anti-IFN-γ antibody or isotype antibody was injected before the 1.2 × 10^3^ TCID_50_ PR8 challenge. Five days after PR8 challenge, the expression of FcγRI, FcγRIIb, FcγRIII, and FcγRIV on alveolar macrophages was determined by flow cytometry. (D and E) A mixture of 1.2 × 10 TCID_50_ PR8 and 2-fold diluted serum obtained from PBS-treated control mice or SV plus CpG/alum-immunized mice was administered to naive mice intranasally. Anti-FcγRIIb antagonistic monoclonal antibody (AT128) or isotype control antibody was injected into these mice and we monitored (D) percentages of initial body weights and (E) survival for the next 15 days. (F and G) A mixture of 1.2 × 10 TCID_50_ PR8 and 2-fold diluted serum obtained from SV plus CpG–ODN-immunized mice was administered to naive mice intranasally. Anti-FcγRIIb agonistic antibody (AT130-2) or isotype control antibody was injected into these mice and we monitored (F) percentages of initial body weights and (G) survival for the next 15 days. (A and B) *n* = 4–5, (C) *n* = 5 for FcγRI, FcγRIII, and FcγRIV, or 15 for FcγRIIb, (D and E) *n* = 8–10, (F and G) *n* = 5 per group. (A, C, D and F) Data are means ± SD. (B) ∗∗p<0.01 between clodronate-loaded liposomes vs. control liposomes group as indicated by comparing Kaplan–Meier curves using the logrank test. (C) ∗∗∗p<0.001 as indicated by Student's ttest. (E) ∗p<0.05 between AT128 plus serum from SV plus CpG/alum-immunized mice vs. isotype control antibody plus serum obtained from SV plus CpG/alum-immunized mice; (G) ∗∗p<0.01 as indicated by comparing Kaplan–Meier curves using the logrank test. See also Figures S7, S8, and S9.

We previously showed that ADCC or ADCP is important for the cross-protection induced by SV plus CpG–ODN because of the interaction between viral-specific non-neutralizing mIgG2b/c and alveolar macrophages (Shibuya et al., 2020). To determine the contribution of alveolar macrophages in conferring cross-protection in SV plus CpG/alum-immunized mice, we depleted them by injecting clodronate-loaded liposomes intranasally into mice immunized with SV plus CpG/alum during the PR8 challenge (Figures 5A and 5B). SV plus CpG/alum-immunized mice treated with clodronate-loaded liposomes exhibited severe weight loss and morbidity compared with mice treated with control liposomes (Figures 5A and 5B). These results suggest that alveolar macrophages are required for the cross-protection afforded by SV plus CpG/alum.

We speculated that IFN-γ modulates the function of alveolar macrophages and particularly enhances ADCC- or ADCP-related functions. We focused on the effects of IFN-γ on the expression of FcγRs on alveolar macrophages. First, we determined the expression of activating FcγRs including FcγRI, FcγRIII, and FcγRIV, and inhibitory FcγRIIb on alveolar macrophages obtained from naive mice and PR8-challenged mice by flow cytometry (Figure S7). In naive mice, we observed the expression of all FcγRs on alveolar macrophages (Figure S7). In addition, on day 5 after the PR8 challenge, the expression of all FcγRs including FcγRIIb was upregulated compared with that in naive mice (Figure S7). Next, to examine the contribution of IFN-γ for the expression of FcγRs, SV plus CpG/alum-immunized mice were treated with anti-IFN-γ antibody during the PR8 challenge, before assessing the expression of FcγRs on alveolar macrophages (Figures 5C and S8). There was no significant difference in the expression of FcγRI, FcγRIII, and FcγRIV between anti-IFN-γ antibody-treated mice and isotype-control antibody-treated mice (Figures 5C and S8). In marked contrast, the expression of FcγRIIb was significantly upregulated in anti-IFN-γ antibody-treated mice compared to isotype-control antibody-treated mice (Figures 5C and S8). To confirm the contribution of IFN-γ for the expression of FcγRIIb, PR8-challenged mice were treated with recombinant IFN-γ intranasally and we analyzed the expression of FcγRIIb on alveolar macrophages (Figure S9). Treatment with recombinant IFN-γ reduced the expression of FcγRIIb significantly (Figure S9). These results indicate that IFN-γ produced by viral-specific CD4^+^ T cells play a crucial role in downregulating the expression of FcγRIIb on alveolar macrophages.

Next, we investigated the contribution of the IFN-γ-mediated downregulation of FcγRIIb in the cross-protection afforded in SV plus CpG/alum-immunized mice (Figures 5D and 5E). Serum from SV plus CpG/alum-immunized mice was mixed with PR8 *in vitro* and transferred to naive mice intranasally and these mice were also administered with anti-FcγRIIb antagonistic monoclonal antibody (AT128) intranasally during the challenge (Figures 5D and 5E). The transfer of serum from SV plus CpG/alum-immunized mice did not induce cross-protection against the PR8 challenge as described in Figure 2A (Figures 5D and 5E). Treatment with AT128 alone did not lead to an improvement in body weight loss or survival (Figures 5D and 5E). However, treatment with both AT128 and the serum from SV plus CpG/alum-immunized mice improved the survival significantly compared with treatment with both isotype control antibody and the serum from SV plus CpG/alum-immunized mice (Figures 5D and 5E). We also examined whether inhibitory signaling via FcγRIIb decreased the cross-protective ability of the serum obtained from SV plus CpG–ODN-immunized mice. The serum obtained from SV plus CpG–ODN-immunized mice was mixed with PR8 *in vitro* and transferred to naive mice intranasally, and these mice were further treated with the anti-FcγRIIb agonistic monoclonal antibody (AT130-2) intranasally during the challenge (Figures 5F and 5G). We observed aggravation of body weight loss and reduction in survival after treatment with the AT130-2 antibody (Figures 5F and 5G). Collectively, these data suggest that the downregulation of FcγRIIb expression due to IFN-γ potentiates the cross-protective activity of cross-reactive non-neutralizing antibodies induced by immunization with SV plus CpG/alum.

### Strong cross-protection of inactivated whole-virion vaccines by adding alum as an adjuvant

**Figure 6:**
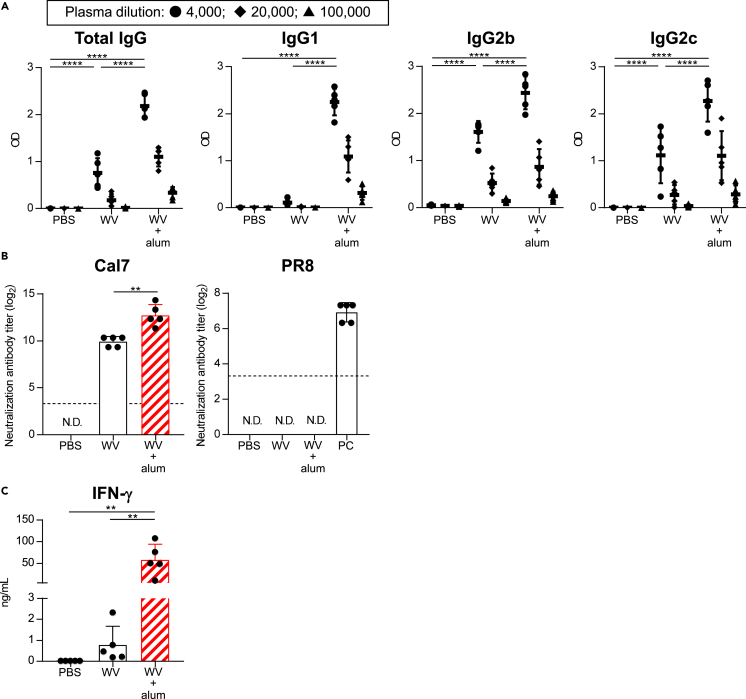
Effects of inactivated whole virion influenza vaccine containing alum Mice were immunized with WV alone and WV plus alum subcutaneously. (A) Levels of WV-specific total mIgG, mIgG1, mIgG2b, and mIgG2c in the plasma were determined after final immunization. We used 4,000- (●), 20,000- (◆), and 100,000- (▲) fold–diluted plasma samples. (B) Neutralization titers against Cal7 and PR8 in the plasma samples obtained from immunized mice were determined using MDCK cells. Plasma samples obtained from PR8-immunized mice were used as a positive control (PC). N.D.: not detected. The dashed line shows the detection threshold for a positive response. (C) Splenocytes obtained from immunized mice were incubated in the presence of WV *in vitro* and the levels of IFN-γ in the supernatants were measured after 3 days. (A–C) *n* = 5 per group. Data are means ± SD. (A and C) ∗∗p<0.01, ∗∗∗∗p<0.0001 as indicated by Tukey’s test. (A) Significant differences were analyzed only in the 4,000-fold-diluted plasma samples. (B) ∗∗p<0.01 as indicated by Student’s t-test.

To verify the broader applicability of our proposed mechanism, we examined the ability of alum to confer cross-protection of WV, again exploring the contribution of IFN-γ for cross-protection. Mice were immunized with WV alone or WV plus alum by using a WV developed from an H1N1 influenza A virus (Cal7) as an antigen. WV plus alum induced significantly higher levels of WV-specific total mIgG, mIgG2b, and mIgG2c than WV alone (Figure 6A). Furthermore, the level of mIgG1 in WV plus alum-immunized mice was also significantly higher than that in WV alone-immunized mice, while WV alone-immunized mice predominantly induced mIgG2b and mIgG2c (Figure 6A). In addition, we did not observe the neutralizing activity against PR8 in both immunized mice, while the plasma samples from WV plus alum-treated group had significantly higher neutralizing activity against Cal7 than mice immunized with WV alone (Figure 6B). The level of IFN-γ secreted by splenocytes in WV plus alum-immunized mice was significantly higher than that in WV alone-immunized mice (Figure 6C). These results suggest that adding alum to WV enhances the production of cross-reactive non-neutralizing antibodies (both mIgG2 and mIgG1) and activation of IFN-γ-producing CD4^+^ T cells against a heterologous virus.

### Synergistic action of IFN-γ and non-neutralizing antibodies for conferring cross-protection in WV plus alum-immunized mice

**Figure 7:**
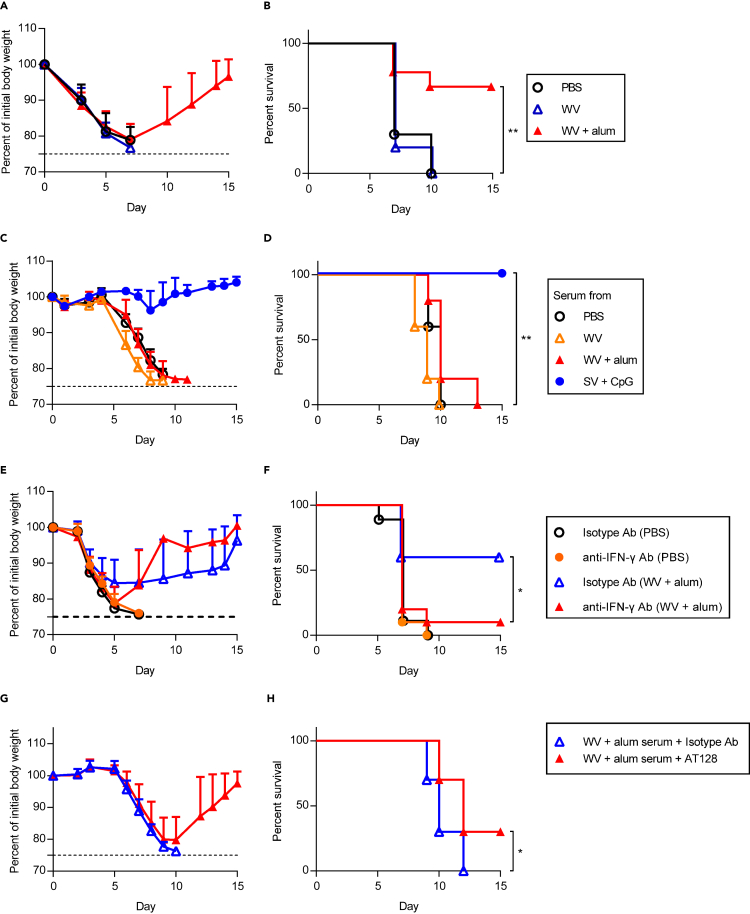
Synergistic effect of non-neutralizing antibodies and IFN-γ for cross-protection induced by inactivated whole virion influenza vaccine with alum (A and B) Ten days after the final immunization, mice were challenged 3.0 × 10^2^ TCID_50_ PR8, we monitored (A) percentages of initial body weights and (B) survival for the next 15 days. (C and D) Mixture of 1.2 × 10 TCID_50_ PR8 and 2 fold diluted serum from PBS-treated control mice, WV alone-, WV plus alum-, SV plus CpG–ODN-immunized mice was transferred into naive mice intranasally. We monitored (C) percentages of initial body weights and (D) survival for the next 15 days. (E and F) After treatment with PBS or immunization with WV plus alum, anti-IFN-γ antibody or isotype antibody was injected into mice before 3.0 × 10^2^ TCID_50_ PR8 challenge. We monitored (E) percentages of initial body weights and (F) survival for the next 15 days. (G and H) A mixture of 1.2 × 10 TCID_50_ PR8 and 2-fold diluted serum obtained from WV plus alum-immunized mice was administered to naive mice intranasally. Anti-FcγRIIb antagonistic monoclonal antibody (AT128) or isotype control antibody was injected into these mice and (G) percentages of initial body weights and (H) survival were monitored for the next 15 days. (A and B) *n* = 9–10, (C and D) *n* = 5, (E, F) *n* = 9–10, (G and H) *n* = 10. (A, C, E and G) Data are means ± SD. (B) ∗∗p<0.01 between WV plus alum group vs WV alone group; (D) ∗∗p<0.01 between serum from SV plus CpG–ODN-immunized mice vs. serum obtained from WV plus alum-immunized mice; (F and H) ∗p<0.05 as indicated by comparing Kaplan–Meier curves using the logrank test.

After final immunization, the same mice were challenged with heterologous PR8 and changes in body weight (Figure 7A) and survival (Figure 7B) were observed. All the mice in PBS-treated control and WV alone-immunized groups died within 10 days after the challenge (Figures 7A and 7B). On the other hand, about 70% of WV plus alum-immunized mice survived, and their body weights recovered after initial weight loss (Figures 7A and 7B). Next, the serum obtained from immunized or PBS-treated control mice was mixed with PR8 *in vitro* and transferred into naive mice intranasally (Figures 7C and 7D). We used plasma samples from SV plus CpG–ODN-immunized mice as a positive control as described above (Figures 7C and 7D). Neither mice group that received the plasma samples from WV alone-immunized mice and WV plus alum-immunized mice conferred cross-protection, although the plasma sample from SV plus CpG–ODN-immunized mice conferred cross-protection (Figures 7C and 7D). To examine the contribution of IFN-γ for WV plus alum-induced cross-protection, mice immunized with WV plus alum were treated with an anti-IFN-γ neutralizing antibody during the PR8 challenge (Figures 7E and 7F). We observed severe weight loss and morbidity in WV plus alum-immunized mice after the treatment with the anti-IFN-γ antibody compared with the isotype-control antibody (Figures 7E and 7F). Next, serum from WV plus alum-immunized mice was mixed with PR8 *in vitro* and transferred to naive mice intranasally and these mice were treated with AT128 intranasally during the challenge as before (Figures 7G and 7H). AT128 significantly improved the survival of WV plus alum-immunized mice compared with isotype control antibody treatment (Figures 7G and 7H). These data suggest that downregulation of FcγRIIb due to IFN-γ potentiates the cross-protective activity of cross-reactive non-neutralizing antibodies induced by immunization with WV plus alum in the same way as with SV plus CpG/alum.

## Discussion

We previously showed that CpG–ODN, but not alum, are optimal adjuvants for conferring cross-protection to influenza by predominantly inducing viral-specific mIgG2b/c (Shibuya et al., 2020). Here, we specifically focused on the difference between the combined use of CpG–ODN and alum with the individual use of CpG–ODN. We showed that SV plus CpG/alum confers stronger cross-protection against a heterologous virus challenge than SV plus CpG–ODN (Figures 2A–2E), although antibodies induced by SV plus CpG/alum, in which both non-neutralizing mIgG1 and mIgG2b/c are induced against the heterologous virus, did not confer cross-protection (Figures 3A, 3B and S3). In contrast, antibodies induced by SV plus CpG–ODN, in which non-neutralizing mIgG2b/c against the heterologous virus are predominantly induced, conferred superior cross-protection (Figures 3A, 3B and S3). In line with our previous findings (Shibuya et al., 2020), we here showed the competitive suppressive effects of cross-reactive non-neutralizing mIgG1 on the cross-protective activity of cross-reactive non-neutralizing mIgG2 (Figures 3C and 3D). Therefore, the lack of cross-protection by antibodies induced by SV plus CpG/alum resulted from the enhanced production of cross-reactive non-neutralizing mIgG1 by adding alum to CpG–ODN.

We also demonstrated that IFN-γ secreted by CD4^+^ T cells acts as an effector molecule to confer cross-protection alongside cross-reactive non-neutralizing mIgG (Figure 4). Furthermore, we provide evidence for the mechanism involved; downregulation of FcγRIIb on alveolar macrophages. The IFN-γ secreted by CD4^+^ T cells nullified the FcγRIIb-mediated suppressive effect of cross-reactive non-neutralizing mIgG and potentiated the cross-protective activity of cross-reactive non-neutralizing mIgG, following the strong cross-protection of SV plus CpG/alum (Figures 5C–5G). Mouse IgG2b/c bind all FcγRs, while mIgG1 binds only FcγRIIb and FcγRIII (Beutier et al., 2017). Therefore, the ability of mIgG1 to induce ADCC/ADCP is lower than that of mIgG2b/c isotypes (Huber et al., 2006; Van den Hoecke et al., 2017; Watanabe et al., 2019). In addition, the binding affinity of mIgG1 to FcγRIIb is about 10 times stronger than that of mIgG2c in mice (Beutier et al., 2017), indicating that the inhibitory impact of FcγRIIb will be stronger with mIgG1 than mIgG2b/c antibodies. Therefore, the FcγRIIb-mediated inhibition by cross-reactive non-neutralizing mIgG1 might be a mechanism to suppress cross-reactive non-neutralizing mIgG2b/c-mediated ADCC or ADCP in SV plus CpG/alum-immunized mice. In fact, we found that the FcγRIIb-mediated inhibitory signaling induced by anti-FcγRIIb agonistic antibody nullifies the cross-protective activity of mIgG2b/c-predominant antibodies induced by SV plus CpG–ODN (Figures 5F and 5G). In addition, our results showed that IFN-γ improves the cross-protective activity of not only the serum obtained from SV plus CpG/alum-immunized mice but also that obtained from SV plus alum-immunized mice (Figure S6), indicating that IFN-γ might also improve not only mIgG2b/c- but also mIgG1-mediated ADCC or ADCP by downregulating the expression of FcγRIIb. Therefore, on the basis of our results, we postulate that IFN-γ secreted by CD4^+^ T cells plays a central role in changing the suppressive effect of mIgG1 to improve cross-protection by downregulating the expression of FcγRIIb. The precise contribution of FcγRIIb in cross-protection against influenza vaccines has remained unclear, although activating FcγR-mediated effector functions have been extensively studied (Bournazos et al., 2020; DiLillo et al., 2014, 2016). In the treatment of cancer with direct targeting antibodies, the suppressive effect of FcγRIIb is a well-documented problem (Clynes et al., 2000) and several therapeutic strategies to overcome it have been suggested (Roghanian et al., 2018). For example, antibody isotype selection and engineering have been implemented to modulate the binding affinity of the therapeutic antibody toward activating FcγRs and away from FcγRIIb (Arce Vargas et al., 2017; Stopforth et al., 2016). In addition, antagonistic antibodies specific for FcγRIIb have been shown to improve the potential of direct targeting anti-cancer therapeutic antibodies (Roghanian et al., 2015). Therefore, we suggest the necessity to elucidate more precise contributions of FcγRIIb and activating FcγRs expressed on alveolar macrophages for conferring cross-protection of influenza vaccines. For example, it is unclear how IFN-γ suppresses the expression of FcγRIIb during a viral infection. It also remains unclear why the expression of activating FcγRs is not changed by IFN-γ, because it is reported that the expression of activating FcγRs is upregulated in response to IFN-γ stimulation in macrophages (Shi et al., 2015). In addition, further investigation is needed to determine whether IFN-γ stimulation modulates the FcγRIIb-mediated inhibitory signaling and activating FcγR-mediated activating signaling, because a functional cross-talk between FcγRs and the IFN-γ receptor has been reported (Bezbradica et al., 2014). Moreover, the mechanism of synergy between antibodies, and CD4^+^ and CD8^+^ T cells needs to be investigated further.

In a mouse model of influenza virus infection, the important contribution of viral-specific T cells has been clearly established (Jansen et al., 2019; Sridhar, 2016). In particular, viral-specific CD8^+^ T cells recognize conserved viral epitopes located on MHC class I and act as effector T cells by killing viral-infected cells (Budimir et al., 2012; Furuya et al., 2010; Guo et al., 2011; Laidlaw et al., 2013). Nevertheless, viral-specific CD8^+^ T cells did not appear to function as effector cells for conferring cross-protection in SV plus CpG/alum immunized mice in this study (Figures 3G and 3H). We speculate that viral-specific CD8^+^ T cells might have been poorly induced in our experiment, because a small amount of SV was used as an antigen. In contrast, CD4^+^ T cells play multiple roles in the development of acquired immunity including the induction of antigen-specific IgG with high affinity, and the activation and maintenance of antigen-specific CD8^+^ T cells. In addition, viral-specific CD4^+^ T cells provide cross-protection as effector cells in specific situations via different mechanisms including direct cytotoxicity and contribution to B-cell activation, although the precise contribution and mechanism remains unclear (Devarajan et al., 2016, 2018; Juno et al., 2017; McKinstry et al., 2012). For example, some studies have shown that viral-specific CD4^+^ T cells with cytotoxic activity eliminate viral-infected cells directly via IFN-γ and perforin production (Brown et al., 2012). However, the contribution of direct killing by CD4^+^ T cells to cross-protection was evidently not substantial in our study, because CD4^+^ T cells alone were not sufficient for conferring cross-protection (Figures3I and 3J). We here showed that viral-specific CD4^+^ T cells play a crucial role as IFN-γ producers in conferring cross-protection to SV plus CpG/alum-immunized mice. Nonetheless, the improvement in survival following treatment with both an anti-FcγRIIb antagonistic monoclonal antibody (AT128) and serum obtained from SV plus CpG/alum-immunized mice was modest, albeit significant, upon PR8 challenge (Figures 5D and 5E), while treatment with both recombinant IFN-γ and serum obtained from SV plus CpG/alum-immunized mice strongly improved survival (Figures 4F and 4G), suggesting additional roles played by IFN-γ in conferring cross-protection. Toward this point, in addition to the downregulation of FcγRIIb expression induced by IFN-γ, our results also showed that IFN-γ produced by CD4^+^ T cells contributed to the recruitment of further CD4^+^ T cells (Figure 4C). Recent reports have shown that IFN-γ induces the production of Th1-type chemokines CXC-chemokine ligand 9 (CXCL9) and CXCL10 from the respiratory epithelium (den Hartog et al., 2020). In addition, several reports have shown that IFN-γ-producing CD4^+^ T cells induce CXCL9 and CXCL10 expression in the lung, and induce the migration of CD4^+^ Th1 cells to inflammatory sites via their cognate receptor CXCR3 in mice (Gocher et al., 2021; Nakanishi et al., 2009; Strutt et al., 2010). Therefore, we speculate that IFN-γ from CD4^+^ T cells enhances the infiltration of CD4^+^ T cells into BALF and lung tissue via CXCL9 and CXCL10. This increase in CD4^+^ T cells infiltrating into the lung might also be important for conferring cross-protection in our model, because the large amount of IFN-γ produced by the higher number of CD4^+^ T cells might further augment the downregulation of FcγRIIb on alveolar macrophages. In general, CD4^+^ T cells induced by prior seasonal influenza virus infection play a crucial role in improving the clinical outcome of influenza virus infection in humans (Wilkinson et al., 2012). Therefore, the potential of CpG–ODN plus alum as adjuvants to enhance IFN-γ-producing CD4^+^ T cells may be important to consider in designing optimal vaccines for humans. Clearly, further investigation is needed to determine the role of IFN-γ-producing CD4^+^ T cells in conferring cross-protection, especially their synergistic effects in association with antibodies, in humans.

Alum-adjuvanted WV is already licensed for use in adults as a vaccine against the H5N1 influenza virus in Japan (Nakayama et al., 2012). Consistent with the results pertaining to the effect of SV plus CpG/alum, we showed that WV plus alum confers superior cross-protection compared with WV alone, although antibodies alone induced by WV plus alum did not confer cross-protection (Figures 6, 7A, and 7B). Furthermore, we observed the synergistic actions of cross-reactive non-neutralizing antibodies induced by WV plus alum and IFN-γ, and the importance of decreased FcγRIIb expression for cross-protection in WV plus alum (Figures 7C–7H). Therefore, we speculate that the decrease of FcγRIIb expression by IFN-γ plays a pivotal role in the cross-protection induced by WV plus alum akin to that seen with SV plus CpG/alum.

In this study, we focused on the cross-protection against heterologous virus challenge *in vivo*. We previously showed that the level of neutralizing antibodies against a homologous virus *in vitro* correlates with the protective activity against homologous virus challenge in mice (Shibuya et al., 2020). Therefore, SV plus CpG/alum and WV plus alum might also strongly protect against homologous virus challenge *in vivo*, because they induce stronger neutralizing antibodies against the homologous virus *in vitro* (Figures 1C and 6B).

The combination of CpG–ODN and alum are expected to be used in humans because CpG–ODN are already approved as an adjuvant for hepatitis B virus vaccine and alum is widely used as an adjuvant in many important human vaccines (HogenEsch et al., 2018; Schillie et al., 2018). However, it remains unclear how the combination of CpG–ODN and alum enhances immune responses, especially IFN-γ-producing CD4^+^ T cells. Alum can adsorb antigens and is suitable for delayed release, leading to the enhancement of antigen persistence and prolonged release, an effect referred to as the “depot effect” (HogenEsch et al., 2018). In our composite adjuvants, alum might adsorb not only SV but also CpG–ODN and the prolonged release of CpG–ODN might also result in enhanced immune responses. In addition, recent studies have suggested the possibility that alum induces the production of damage-associated molecular patterns (DAMPs) following the effective induction of adaptive immunity (HogenEsch et al., 2018). The combinatorial effects of DAMP-inducing adjuvants and CpG–ODN have also been reported (Hayashi et al., 2018). Therefore, CpG–ODN and alum might induce different but synergistic innate immune responses to enhance acquired immune responses. Furthermore, the synergistic effects of adjuvants using a combination of another TLR agonists and alum for the development of vaccines that are more potent and offer broader protection against influenza viruses require investigation because a rational combination of adjuvants induces not only antigen-specific antibodies but also antigen-specific CD4^+^ and CD8^+^ T cells to elicit broader cross-protection (Kasturi et al., 2011; Napolitani et al., 2005).

### Limitations of the study

For vaccine development, it is important to understand the differences in immune responses between mice and humans, in order to more faithfully translate the results obtained in mice as predictions for humans. For example, antibody isotypes, FcγR-binding affinities and expression profiles of FcγRs differ between mice and humans and should be taken into careful consideration when inferring likely effects in humans. Although we have not demonstrated effects with human isotypes, FcγRs, cells or subjects, we feel the concepts derived from our study bear scrutiny for translation. Like the mouse IgG2 studied here, human IgG1 and IgG3 can bind to human activating FcγRs and have strong ADCC activity (Bruhns et al., 2009). Indeed, HA-specific IgG1 and IgG3 may be the critical isotypes for ADCC-mediated protection in humans (Vanderven et al., 2017). In contrast, human IgG2 and IgG4, like mouse IgG1, interact less strongly with FcγRs in general and elicit less powerful ADCC. Therefore, virus-specific IgG2 and IgG4 might interfere with the cross-protective effects of virus-specific-IgG1 and IgG3 in humans, much as the mIgG1 was shown to interfere with mIgG2 here. Furthermore, the expression levels and the expression patterns of FcγRs in several types of immune cells are different between mice and humans. For example, the expression of FcγRIIb on monocytes and neutrophils, including alveolar macrophages, is very low in humans and higher in mice. Mice in which human FcγRs replace their murine counterparts, as generated previously (Gillis et al., 2017; Smith et al., 2012) would help address these issues as they express human FcγRs that bind human IgG isotypes in a physiologically relevant manner and in a pattern that more closely recapitulates the expression pattern seen in humans. Such mice can aid translation. For example, Bournazos et al. recently developed anti-influenza IgG monoclonal antibodies using mice of this nature and showed that conventional dendritic cells express the human inhibitory FcγRIIb in the lung (Bournazos et al., 2020). However, even these mice do not fully recapitulate human FcγR expression patterns, expressing far higher levels of FcγRIIb on monocytes than humans. Therefore, further study is needed to overcome the limitations of the differences in immune response between mice and humans for translating our concept for human use in the future. Nevertheless, we feel that the proof of concept has been established in the mice in our study, with the relevant implications for humans outlined.

## STAR★Methods

### Key resources table

**Table:** 

REAGENT or RESOURCE	SOURCE	IDENTIFIER
**Antibodies**		

Goat polyclonal anti-mouse IgG with HRP	Merck Millipore	Cat# AP503P RRID: AB_805355
Goat polyclonal anti-mouse IgG1 with HRP	SouthernBiotech	Cat# 1070-05; RRID: AB_2650509
Goat polyclonal anti-mouse IgG2b with HRP	SouthernBiotech	Cat# 1090-05; RRID: AB_2794521
Goat polyclonal anti-mouse IgG2c with HRP	SouthernBiotech	Cat# 1079-05; RRID: AB_2794466
Anti-mouse CD16/CD32 (clone: 93)	BioLegend	Cat# 101302; RRID: AB_312801
BV421 anti-mouse CD45 (clone: 30-F11)	BioLegend	Cat# 103134; RRID: AB_2562559
FITC anti-mouse Ly6G (clone: 1A8)	BioLegend	Cat# 127605; RRID: AB_1236488
BV785 anti-mouse CD11b (clone: M1/70)	BioLegend	Cat# 101243; RRID: AB_2561373
APC anti-mouse CD3 (clone: 145-2C11)	BioLegend	Cat# 100312; RRID: AB_312677
PE/Cy7 anti-mouse CD4 (clone: RM4-5)	BioLegend	Cat# 100528; RRID: AB_312729
PerCP anti-mouse CD8a (clone: 53-6.7)	BioLegend	Cat# 100731; RRID: AB_893427
APC/Cy7 anti-mouse CD11c (clone: N418)	BioLegend	Cat# 117324; RRID: AB_830649
APC anti-mouse Siglec-F (clone: REA798)	Miltenyi Biotec	Cat# 130-112-333 RRID: AB_2653441
PE anti-mouse FcγRI (CD64; clone: X54-5/7.1)	BioLegend	Cat# 139303; RRID: AB_10613467
PE mouse IgG1 κ isotype control (clone: MOPC-21)	BioLegend	Cat# 400111
PE anti-mouse FcγRIIb (CD32b; clone: AT130-2)	Invitrogen	Cat# 12-0321-82; RRID: AB_2572557
PE mouse IgG2a κ isotype control (clone: eBM2a)	Invitrogen	Cat# 12-4724-82; RRID: AB_470064
PE anti-mouse FcγRIII (CD16; clone: 275003)	R&D Systems	Cat# FAB19601P-100 RRID: AB_2246942
PE rat IgG2a isotype control (clone: 54447)	R&D Systems	Cat# IC006P RRID: AB_357256
PE anti-mouse FcγRIV (CD16.2; clone: 9E9)	BioLegend	Cat# 149503; RRID: AB_2565810
PE american hamster IgG isotype control (clone: HTK888)	BioLegend	Cat# 400907 RRID: AB_326593
Rat IgG2b κ isotype control (clone: RTK4530)	BioLegend	Cat# 400672
Rat IgG2a isotype control (clone: 2A3)	BioXcell	Cat# BE0089; RRID: AB_1107769
Anti-IFN-γ antibody (clone: XMG1.2)	BioXcell	Cat# BE0055; RRID: AB_1107694
Rat IgG1 isotype control (clone: HRPN)	BioXcell	Cat# BE0088; RRID: AB_1107775
Antagonistic monoclonal antibody against FcγRIIb (clone: AT128)	Dr. Mark S. Cragg, University of Southampton	N/A
Mouse IgG1 isotype control (clone: MOPC-21)	BioXcell	Cat# BE0083; RRID: AB_1107784
Agonistic monoclonal antibody against FcγRIIb (clone: AT130-2)	Dr. Mark S. Cragg, University of Southampton	N/A
Mouse IgG2a isotype control (clone: C1.18.4)	BioXcell	Cat# BE0085; RRID: AB_1107771
BV711 anti-mouse IFN-γ (clone: XMG1.2)	BioLegend	Cat# 505836; RRID: AB_2650928
PE-Cy7 anti-mouse IL-13 (clone: eBio13A)	Thermo Fisher Scientific	Cat# 25-7133-80; RRID: AB_2573530
FITC anti-mouse CD4 (clone: GK1.5)	BioLegend	Cat# 100406; RRID: AB_312691
BV605 anti-mouse CD8 (clone: 53-6.7)	BioLegend	Cat# 100744; RRID: AB_2562609
AF647 anti-mouse CD45 (clone: 30-F11)	BioLegend	Cat# 103124; RRID: AB_493533
PE anti-mouse CD3 (clone: 145-2C11)	BioLegend	Cat# 100308; RRID: AB_312673

**Bacterial and virus strains**		

Ether-treated hemagglutinin-antigen-enriched virion-free split vaccine (SV) from H1N1 influenza virus (strain: A/California/7/2009 (Cal7))	Dr. YasuyukiGomi, The Research Foundation for Microbial Diseases of Osaka University	N/A
Formalin-inactivated whole-virion vaccine (WV) from H1N1 influenza virus (strain: Cal7)	Dr. YasuyukiGomi, The Research Foundation for Microbial Diseases of Osaka University	N/A
H1N1 influenza virus (strain: A/Puerto Rico/8/34 (PR8))	Dr. YasuyukiGomi, The Research Foundation for Microbial Diseases of Osaka University	N/A
H1N1 influenza virus (strain: Cal7)	Dr. Hideki Asanuma, National Institute of Infectious Diseases	N/A

**Chemicals, peptides, and recombinant proteins**		

Recombinant HA proteins derived from Cal7	Kawai et al., 2020	N/A
Recombinant HA proteins derived from PR8	Kawai et al., 2020	N/A
Alhydrogel Adjuvant 2%	InvivoGen	Cat# vac-alu-250
Block Ace	DS Pharma Biomedical	Cat# UKB80
Tetramethyl Benzidine	Nacalai Tesque	Cat# 05299-54
Receptor-destroying Enzyme (RDE) (II)	Denka Seiken	Cat# 340016
Amido Black	Nacalai Tesque	Cat# 01927-92
Guinea Pig Red Blood Cells	Japan Bio Serum	Cat# 035-00012
7-amino-actinomycin D (7-AAD)	BioLegend	Cat# 420404
Recombinant Mouse IFN-γ	BioLegend	Cat# 575306
Clophosome®-A-Clodronate Liposomes (anionic)	FormuMax Scientific Inc.	Cat# F70101C-A
Plain Control Liposomes (Anionic)	FormuMax Scientific Inc.	Cat# F70101-A
Protein Transport Inhibitor Cocktail	Thermo Fisher Scientific	Cat# 00-4980-93
Brefeldin A	Sigma-Aldrich	Cat# B6542
Collagenase Type IV	Gibco	Cat# 17104-019
Deoxyribonuclease 1	Wako	Cat# 047-26771
HiTrap Protein G HP	GE Healthcare	Cat# 17040403
HiTrap Protein A HP	GE Healthcare	Cat# 29048576
Fixable Viability Dye eFluor 780	Thermo Fisher Scientific	Cat# 65-0865-14

**Critical commercial assays**		

CD4+ T cell Isolation Kit	Miltenyi Biotec	Cat# 130-104-454
Expi293™ GnTI- Expression System Kit	Thermo Fisher Scientific	Cat# A39250
IFN-γ ELISA Kit	BioLegend	Cat# 430801
IL-13 ELISA Kit	R&D Systems	Cat# DY413-05
Pierce BCA Protein Assay Kit	Thermo Fisher Scientific	Cat# 23225
Fixation/Permeabilization Solution Kit with BD GolgiPlug	BD Biosciences	Cat# 555028

**Experimental models: Cell lines**		

Madin-Darby Canine Kidney (MDCK) cells	The Research Foundation for Microbial Diseases of Osaka University	N/A
GK1.5 (Anti-CD4 antibody)	ATCC	Cat# TIB-207
53-6.72 (Anti-CD8a antibody)	ATCC	Cat# TIB-105

**Experimental models: Organisms/strains**		

C57BL/6JJmsSlc	Japan SLC	N/A

**Oligonucleotides**		

CpG–ODN (CpG K3: 5′-atcgactctcgagcgttctc-3′)	Gene Design	Cat# CN-65003

**Software and algorithms**		

GraphPad Prism 7.03	GraphPad Software	Version 7.03
Flowjo Software	TreeStar	N/A

**Other**		

Microplate Reader (Power Wave HT)	BioTek	N/A
NovoCyteFlow Cytometer	ACEA Bioscience	N/A
Attune NxT Flow Cytometer	Thermo Fisher Scientific	N/A
Gentle MACS Dissociator	Miltenyi Biotech	N/A
AKTA pure 25 L1	GE Healthcare	N/A

### Resource availability

#### Lead contact

Further information and requests for resources and reagents should be directed to, and will be fulfilled by, the Lead Contact, Yasuo Yoshioka (y-yoshioka@biken.osaka-u.ac.jp).

#### Materials availability

All reagents used in this study will be made available upon reasonable request to the lead contact.

### Experimental model and subject details

#### Mice

Male C57BL/6J mice (6 to 7 weeks of age) were purchased from SLC (Shizuoka, Japan). Mice were housed in a room with a 12:12-h light:dark cycle and had unrestricted access to food and water. All animal experiments were conducted in accordance with the guidelines of Osaka University for the ethical treatment of animals and were approved by the Animal Care and Use Committee of the Research Institute for Microbial Diseases, Osaka University, Japan (protocol number, BIKEN-AP-H26-11-0).

### Method details

#### Split vaccine, inactivated whole-virion vaccine, and influenza viruses

Ether-treated hemagglutinin-antigen-enriched virion-free split vaccine (SV) from H1N1 influenza virus (strain: A/California/7/2009 (Cal7)), formalin-inactivated whole-virion vaccine (WV) from H1N1 influenza virus (strain: Cal7), and H1N1 influenza virus (strain: A/Puerto Rico/8/34 (PR8)) were kindly provided by Dr. YasuyukiGomi (the Research Foundation for Microbial Diseases of Osaka University, Kagawa, Japan). H1N1 influenza virus (strain: Cal7) was kindly provided by Dr. Hideki Asanuma (the National Institute of Infectious Diseases, Tokyo, Japan).

#### Recombinant hemagglutinin (HA) proteins

The amino acid sequences for HA were derived from Cal7 (GenBank accession number: ACV82259.1) and PR8 (GenBank accession number: LC120393.1). Human codon-optimized cDNA of the ectodomain of HA (amino acids 1–523) with a C-terminal hexahistidine tag was cloned into the pcDNA3.1 expression plasmid (Thermo Fisher Scientific, Hampton, NH, USA). The foldon trimerization domain sequence (GYIPEAPRDGQAYVRKDGEWVLLSTFL) from fibritin of bacteriophage T4 was inserted at the C terminal of HA. Secreted soluble recombinant HA was expressed using Expi293 Expression System (Thermo Fisher Scientific) in accordance with the manufacturer’s instructions. The recombinant HA in the supernatant was then purified using an AKTAexplorer chromatography system with a Ni-Sepharose HisTrap FF column (GE Healthcare, Diegem, Belgium) and a Superose 6 Increase 10/300 GL column (GE Healthcare).

#### Immunization and virus infection

CpG–ODN (CpG K3: 5′-atcgactctcgagcgttctc-3′) was purchased from Gene Design (Osaka, Japan). Alhydrogel adjuvant 2% as alum was purchased from InvivoGen (San Diego, CA, USA). Mice were subcutaneously immunized at the base of the tail with SV (0.5 μg HA/mouse) alone or SV (0.5 μg HA/mouse) with 50 μg of CpG–ODN, 250 μg of alum, or 50 μg of CpG–ODN plus 250 μg of alum in 50 μL of PBS, two times at 14-day intervals. For the experiments performed using WV, mice were subcutaneously immunized at the base of the tail with WV (1 μg/mouse) alone or WV (1 μg/mouse) with 50 μg of alum in 50 μL of PBS, two times at 21-day intervals. Mice that were subcutaneously immunized with PBS alone were used as control. As a positive control (Figures 1C, 6B, and S1), mice were subcutaneously immunized at the base of the tail with 2 × 10^3^ TCID_50_ of PR8, two times at 14-day intervals. On day 7 after final immunization, plasma samples were collected from mice and stored at −30°C until further use. On day 10 after final immunization, mice were intranasally challenged with 1.2 × 10^3^, 3.0 × 10^2^, or 1.2 × 10 TCID_50_ of PR8 in 30 μL of PBS under anesthesia (Tamura et al., 1988). Body weights and survival of the challenged mice were monitored for 15 days post-challenge. The humane endpoint was set at 25% body weight loss relative to the initial body weight at the time of infection. We defined the day that mice had less than 75% body weight to initial body weight as the day of death.

#### Detection of antigen-specific antibodies

The levels of SV-, recombinant HA-, or WV-specific antibodies in the plasma samples were determined using enzyme-linked immunosorbent assay (ELISA). ELISA plates were coated with 10 μg/mL SV in PBS, 1 μg/mL recombinant HA in 0.1 M sodium carbonate buffer (pH 9.6), or 10 μg/mL WV in PBS overnight at 4°C. The plates were incubated with Block Ace (DS Pharma Biomedical, Osaka, Japan), and then plasma samples were added to the antigen-coated plates. After washing the plates with PBS containing 0.05% Tween 20, the plates were incubated with horseradish peroxidase-conjugated goat anti-mouse IgG antibody (Merck Millipore, Darmstadt, Germany), IgG1 antibody (SouthernBiotech, Birmingham, AL, USA), IgG2b antibody (SouthernBiotech), or IgG2c antibody (SouthernBiotech). After washing, tetramethyl benzidine (Nacalai Tesque, Kyoto, Japan) was added and the color reaction was stopped by adding 2 N H_2_SO_4_, and the absorbance was measured at OD_450–570_ using a microplate reader (Power Wave HT, BioTek, Winooski, VT, USA).

#### Neutralization assay and hemagglutination inhibition (HI) assay

Plasma samples were treated with receptor-destroying enzyme (RDE) (II) (Denka Seiken, Tokyo, Japan) for 18 h at 37°C and then heated at 56°C for 1 h to deactivate the enzyme. Serially diluted RDE (II)-treated plasma samples were incubated with Cal7 or PR8 at final concentrations of 10^2^ TCID_50_ at 37°C for 30 min. MDCK cells were incubated with the mixtures at 37°C for 3 days. After the cells were treated with 0.1% amido black (Nacalai Tesque) and 0.1N NaOH, OD_630_ was measured using a microplate reader. For analysis of HItiters, after RDE (II)-treated plasma samples were incubated with guinea pig red blood cells for 1 h, the serially diluted plasma samples were incubated with Cal7 or PR8 at final concentrations of 8 HAU/50 μL. The HI titer was determined based on the dilution rate of the plasma samples, which show non-agglutinated red blood cells.

#### Cytokine production from splenocytes

On day 7 after final immunization, splenocytes (1 × 10^6^ cells) collected from spleens were treated with or without SV or WV (final concentration, 10 μg/mL) for 3 days at 37°C in 96-well plates. After incubation, the concentrations of IFN-γ and IL-13 in the supernatants were measured by using ELISA (IFN-γ: BioLegend, San Diego, CA, USA; IL-13: R&D Systems, Minneapolis, MN, USA) in accordance with the manufacturer’s instructions.

#### Bronchoalveolar lavage fluid (BALF)

On day 5 after influenza virus challenge, BALF was collected by lavaging the lung with 1.2 mL of PBS. The BALF was centrifuged at 600×*g* for 5 min and cell pellets were collected for flow cytometric analysis. The supernatant was used for the measurement of the virus titer. Virus titers were determined by infection of MDCK cells as described above.

#### Flow cytometry

To determine the number of CD4^+^ and CD8^+^ T cells in the collected cells from BALF, we incubated the cells with anti-mouse CD16/CD32 antibody (clone: 93; BioLegend), BV421 anti-mouse CD45 antibody (clone: 30-F11; BioLegend), FITC anti-mouse Ly6G antibody (clone: 1A8; BioLegend), BV785 anti-mouse CD11b antibody (clone: M1/70; BioLegend), APC anti-mouse CD3 antibody (clone: 145-2C11; BioLegend), PE/Cy7 anti-mouse CD4 antibody (clone: RM4-5; BioLegend), and PerCP anti-mouse CD8 antibody (clone: 53-6.7; BioLegend). We defined CD4^+^ or CD8^+^ T cells in BALF as CD45^+^ Ly6G^-^ CD11b^-^ CD3^+^ CD4^+^ or CD45^+^ Ly6G^-^ CD11b^-^ CD3^+^ CD8^+^ cells, respectively. To detect alveolar macrophages in BALF, we incubated the cells with BV421 anti-mouse CD45 antibody (clone: 30-F11; BioLegend), APC/Cy7 anti-mouse CD11c antibody (clone: N418; BioLegend), APC anti-mouse Siglec-F antibody (clone: REA798; Miltenyi Biotec, Gladbach, Germany), and 7-amino-actinomycin D (7-AAD; BioLegend). We defined alveolar macrophages in BALF as CD45^+^ CD11c^+^ Siglec-F^+^ 7-AAD^-^ cells. To determine the expression of FcγRI, FcγRIIb, FcγRIII, and FcγRIV on alveolar macrophages, we used PE anti-mouse FcγRI antibody (CD64; clone: X54-5/7.1; BioLegend), PE mouse IgG1 κ as the isotype control for anti-mouse FcγRI antibody (clone: MOPC-21; BioLegend), PE anti-mouse FcγRIIb antibody (CD32b; clone: AT130-2; invitrogen), PE mouse IgG2a κ as isotype control for anti-mouse FcγRIIb antibody (clone: eBM2a; invitrogen), PE anti-mouse FcγRIII antibody (CD16; clone: 275003; R&D Systems), PE Rat IgG2a as isotype control for anti-mouse FcγRIII antibody (clone: 54447; R&D Systems), PE anti-mouse FcγRIV antibody (CD16.2; clone: 9E9; BioLegend), or PE american Hamster IgG as isotype control for anti-mouse FcγRIV antibody (clone: HTK888; BioLegend). Flow cytometric analysis was performed using NovoCyte Flow Cytometer (ACEA Bioscience, San Diego, CA, USA). For evaluating cytokine production from splenocytes by flow cytometry, splenocytes (1-3 x 10^6^ cells) derived from SV plus CpG/alum-immunized mice on day 7 after final immunization were treated with SV (10 μg/mL) and 1:500 diluted protein transport inhibitor cocktail (Thermo Fisher Scientific) for 6 h at 37°C in 96-well plates. After incubation, cells were stained with Fixable Viability Dye eFluor 780 (Thermo Fisher Scientific), FITC anti-mouse CD4 antibody (clone: RM4-5; BioLegend), and BV605 anti-mouse CD8 antibody (clone: 53-6.7; BioLegend), followed by intracellular IFN-γ (clone: XMG1.2, BV711, Biolegend) and IL-13 (clone: eBio13A, PE/Cy7, Thermo Fisher Scientific) staining using a BD Cytofix/Cytoperm™ Fixation/Permeablization Kit in accordance with the manufacturer’s instructions. Flow cytometric analysis was performed using NovoCyte Flow Cytometer (ACEA Bioscience, San Diego, CA, USA) or Attune NxT Flow Cytometer (Thermo Fisher Scientific). Flowjo software (TreeStar, Ashland, OR, USA) was used for analysis.

#### Serum adoptive transfer

On day 7 after final immunization, serum was collected from immunized mice or PBS-treated control mice. Pooled serum sample was mixed with 1.2 × 10 or 6 × 10^2^ TCID_50_ of PR8 *in vitro*, and this mixture was injected into naïve mice intranasally in 30 μL of PBS under anesthesia. Body weights and survival of the challenged mice were monitored. As shown in Figures 4F and 4G, mice were treated with 5 μg of recombinant IFN-γ (BioLegend) intranasally in 30 μL of PBS under anesthesia 5 times on −1, 0, 1, 2, and 3 days after the virus challenge.

#### Adoptive transfer of purified mIgGs

To separate the total mIgG, serum samples were applied to a Protein G column (GE Healthcare) equilibrated with 20 mM phosphate buffer (pH 7) using an Akta explorer chromatography system (GE Healthcare). After being washed with phosphate buffer, the total mIgG was eluted in 100 mM glycine HCl buffer (pH 2.7), and the eluted solution was immediately neutralized with 1M Tris-HCl buffer (pH 9.0). The buffer of the total mIgG solution was exchanged with PBS. For mIgG2 isolation, the serum sample was applied to a Protein A column (GE Healthcare) equilibrated with 100 mM Tris-HCl buffer (pH 8.0) using an Akta explorer chromatography system. mIgG1 was eluted with 100 mM citric acid buffer (pH 5). Next, mIgG2 was eluted with 100 mM citric acid buffer (pH 3), and the buffer of the eluted solution was immediately exchanged with PBS. The amount of purified total mIgG or mIgG2 was quantified using a Pierce BCA Protein Assay Kit (Thermo Fisher Scientific) with a bovine serum albumin standard. To ascertain binding to SV, SV-specific mIgG1, mIgG2b, and mIgG2c in 5 ng of purified total mIgG and purified mIgG2 were detected by ELISA, as described above. For the *in vivo* experiment, 20 μg of purified total mIgG or purified mIgG2 was mixed with 6 TCID_50_ PR8 *in vitro*, and this mixture was injected into naïve mice intranasally in 30 μL of PBS under anesthesia. Body weights and survival of the challenged mice were monitored.

#### Immune cell depletion *in vivo*

To deplete T cells, on days 9 and 13 after final immunization, mice were intraperitoneally injected with 200 μg of anti-CD4 antibody (clone: GK1.5), isotype control antibody for anti-CD4 antibody (clone: RTK4530; BioLegend), 200 μg of anti-CD8 antibody (clone: 53.6.7), isotype control antibody for anti-CD8 antibody (clone: 2A3; BioXcell, West Lebanon, NH). To deplete alveolar macrophages on day 1 after final immunization, mice were intranasally injected with 30 μL of clodronate-loaded liposomes (Clophosome A, FormuMax Scientific Inc., Sunnyvale, CA, USA) or control liposome (FormuMax Scientific Inc.) under anesthesia. On 10 days after the final immunization, mice were intranasally challenged with 1.2 × 10^3^ TCID_50_ of PR8 in 30 μL PBS under anesthesia. Body weights and survival of challenged mice were monitored.

#### Adoptive transfer of CD4^+^ T cells

On day 7 after final immunization, spleens were recovered from mice immunized with SV plus CpG/alum. CD4^+^ T cells from pooled splenocytes were negatively isolated with a CD4^+^ T cell isolation kit (Miltenyi Biotec) according to the manufacturer’s instructions. CD4^+^ T cell purity was determined by flow cytometry (CD3^+^ CD4^+^ cells >95% purification). Immediately after cell preparation, CD4^+^ T cells (5 × 10^6^ cells/mouse) were injected intravenously into naïve recipient mice. Pooled serum sample from SV plus CpG/alum-immunized mice or PBS-treated control mice was mixed with 1.2 × 10 TCID_50_ of PR8 *in vitro*, and this mixture was injected into naïve mice or CD4^+^ T cell-transferred mice intranasally in 30 μL of PBS under anesthesia at 3 h after CD4^+^ T cells adoptive transfer. Body weights and survival of the challenged mice were monitored.

#### Anti-IFN-γ antibody treatment

To neutralize IFN-γ, on day 9 after final immunization, immunized mice or PBS-treated control mice were intraperitoneally injected with 250 μg of anti-IFN-γ antibody (clone: XMG1.2; BioXcell) or 250 μg of isotype control antibody (clone: HRPN; BioXcell). On day 10 after final immunization, mice were intranasally challenged with 1.2 × 10^3^TCID_50_ of PR8 in 30 μL PBS under anesthesia. Body weights and survival of challenged mice were monitored. On day 5 after the challenge, the expression of FcγRs on alveolar macrophages in BALF was determined by flow cytometry.

#### Evaluating *in vivo* IFN-γ-producing cells by flow cytometry

On day 10 after final immunization, mice were intranasally challenged with 1.2 × 10^3^ TCID50 of PR8 in 30 μL of PBS under anesthesia. On day 4 post challenge, 250 μg of Brefeldin A (Sigma-Aldrich, St. Louis, MO, USA) in 500 μL PBS was injected intraperitoneally. Six hours after the Brefeldin A injection, the lung was harvested and digested with Collagenase type IV (200 U/ml) (Thermo Fisher Scientific) and DNase (100 U/ml) (Wako, Saitama, Japan), with 2.5 μg/mL Brefeldin A for 1 h at 37°C, and homogenized using a gentle MACS™ Dissociator (Miltenyi Biotech, Gladbach, Germany). The resulting cells were then stained with Fixable Viability Dye eFluor 780 (Thermo Fisher Scientific), AF647 anti-mouse CD45 antibody (clone: 30-F11; BioLegend), PE anti-mouse CD3 antibody (clone: 145-2C11; BioLegend), and FITC anti-mouse CD4 antibody (clone: RM4-5; BioLegend), and then with intracellular IFN-γ as described above. CD4^+^ T cells were determined as Fixable Viability Dye^-^ CD45^+^ SSC^low^ CD3^+^ CD4^+^ cells.

#### FcγRIIb expression on alveolar macrophages

Naïve mice were challenged with 1.2 × 10^3^ TCID_50_ of PR8 in 30 μL PBS under anesthesia. On day 4 after the challenge, mice were treated with 5 μg of recombinant IFN-γ (BioLegend) intranasally in 30 μL of PBS under anesthesia. On day 5 after the challenge, the expression of FcγRIIb on alveolar macrophages in BALF was determined by flow cytometry as described above.

#### Inhibition and activation of FcγRIIb by antagonistic and agonistic antibodies

Antagonistic monoclonal antibody against FcγRIIb (clone: AT128) and agonistic monoclonal antibody against FcγRIIb (clone: AT130-2) were described previously (Williams et al., 2012). On day 7 after final immunization, serum was collected from PBS-treated control mice or immunized mice. Pooled serum sample was mixed with 1.2 × 10 TCID_50_ of PR8 *in vitro*, and this mixture was injected into naïve mice intranasally in 30 μL of PBS under anesthesia. Mice were treated with 20 μg of AT128, isotype control antibody for AT128 (clone: MOPC-21; BioXcell), AT130-2, or isotype control antibody for AT130-2 (clone: C1.18.4; BioXcell) intranasally in 30 μL of PBS under anesthesia 2 times on –1 and 5 days after the virus challenge.

### Quantification and statistical analysis

#### Statistical analyses

Statistical analyses were performed with Prism (GraphPad Software, San Diego, CA, USA). All data are presented as means with standard deviation (SD). Significant differences were determined by means of Tukey’s test, Student’s *t*-test or Bonferroni’s test. Significant differences in survival were obtained by comparing Kaplan–Meier curves using the log-rank test. A *P* value of <0.05 was considered to indicate statistical significance. All experiments were repeated at least twice.

## Data Availability

• All data reported in this paper will be shared by the lead contact upon request. • This paper does not report original code. • Any additional information required to reanalyze the data reported in this paper is available from the lead contact upon request.
