# Supplementary material for: Synergistic effect of non-neutralizing antibodies and interferon-γ for cross-protection against influenza
Source: iScience. 2021 Sep 15;24(10):103131. doi: 10.1016/j.isci.2021.103131 (PMC8482522; doi:10.1016/j.isci.2021.103131)
Supplement: Document S1. Figures S1–S9 [file mmc1.pdf]

iScience, Volume *24*

Supplemental information

Synergistic effect of non-neutralizing antibodies and interferon-γ for cross-protection against influenza

Meito Shibuya, Shigeyuki Tamiya, Atsushi Kawai, Toshiro Hirai, Mark S. Cragg, and Yasuo Yoshioka

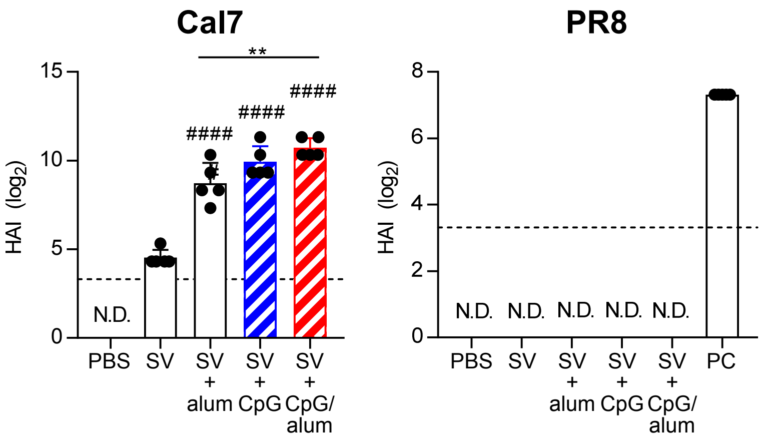

Figure S1. Hemagglutination inhibition (HI) titers, Related to Figure 1. Mice were immunized with SV alone, SV plus alum, SV plus CpG–ODN, or SV plus CpG/alum subcutaneously. Mice administered PBS subcutaneously were considered control mice. Levels of HI titer in the plasma samples of immunized mice were determined. The dashed line shows the detection threshold for a positive response. In the right graph, the plasma samples obtained from PR8-immunized mice were used as a positive control (PC). *n* = 5 per group. Data are means ± SD. N.D.: not detected. $^{####}$*P* <0.0001 vs. SV alone group; ***P* <0.01 as indicated by Tukey's test.

Figure S1. Shibuya et al.

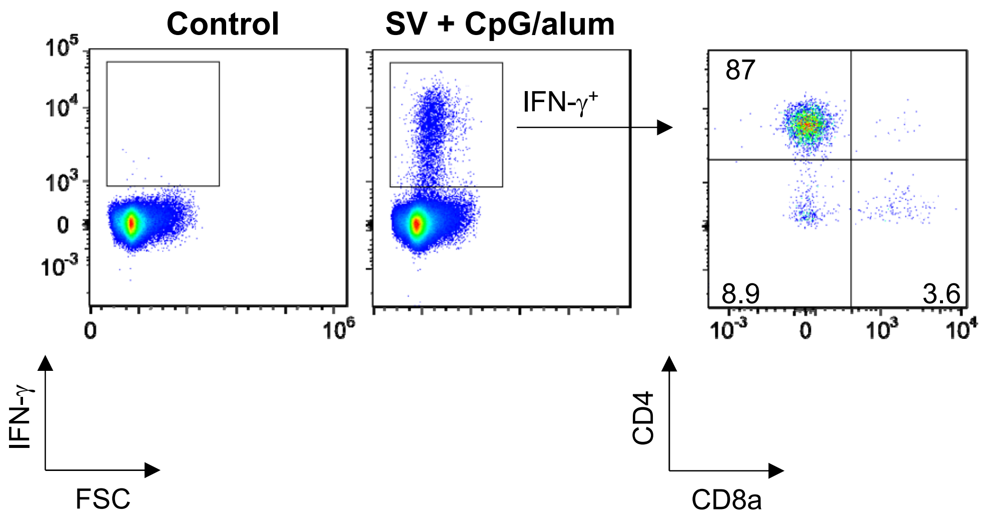

Figure S2. Gating strategy for identifying IFN-γ-producing cells, Related to Figure 1. Splenocytes obtained from SV plus CpG/alum-immunized mice were incubated in the presence of SV with protein transport inhibitor cocktail for 24 h in vitro and the intracellular levels of IFN-γ in live cells were evaluated by flow cytometry. Representative flow cytometry plots from data presented in Figure 1E are shown.

Figure S2. Shibuya et al.

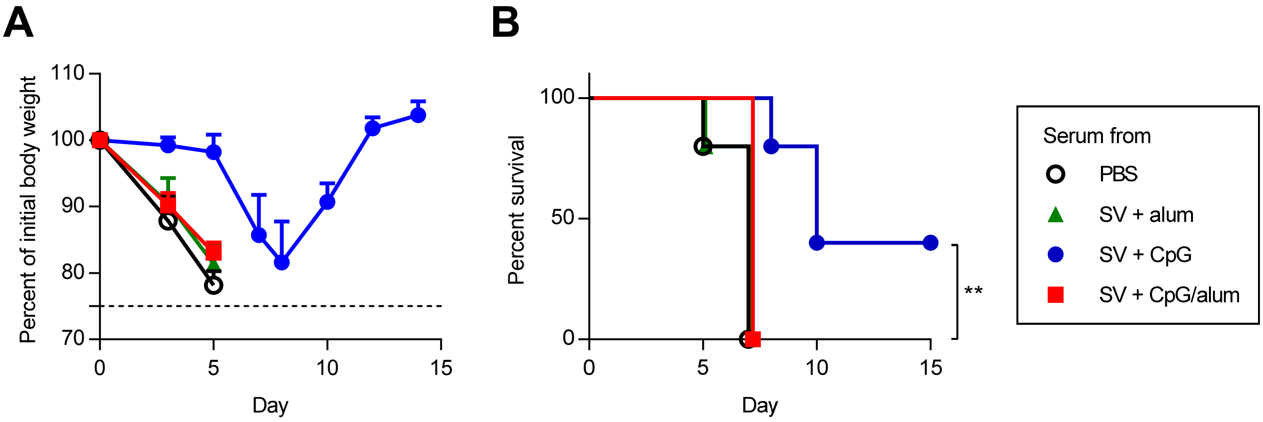

Figure S3. Serum transfer, Related to Figure 3. A mixture of 6 × 10^2 $TCID_{50}$ PR8 and 2-fold diluted serum obtained from PBS-treated control mice, SV plus alum-, SV plus CpG–ODN-, or SV plus CpG/alum-immunized mice was administered to naïve mice intranasally. We monitored **(A)** percentages of initial body weights and **(B)** survival for the next 15 days. **(A, B)** *n* = 5 per group. **(A)** Data are means ± SD. **(B)** $^{**}P$ <0.01 between SV plus CpG–ODN group vs. SV plus CpG/alum group as indicated by comparing Kaplan-Meier curves using the log-rank test.

Figure S3. Shibuya et al.

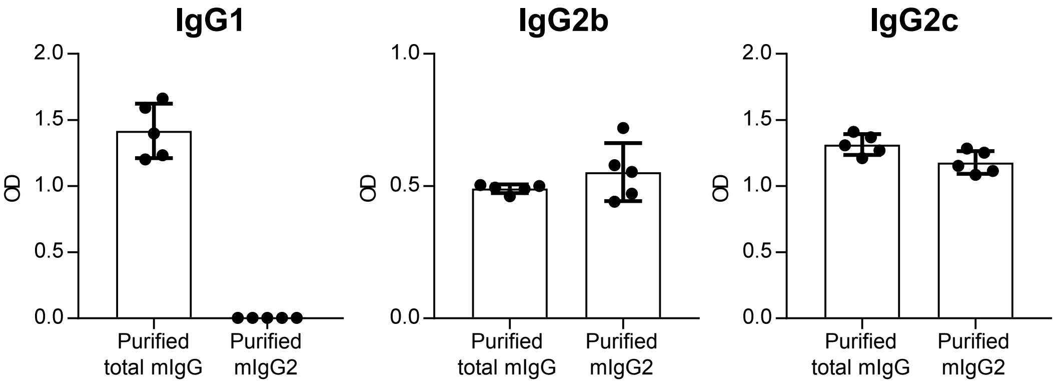

Figure S4. Isolation of mIgG2, Related to Figure 3. Total mIgG and mIgG2 were purified from the serum of SV plus CpG/alum-immunized mice. Levels of SV-specific mIgG1, mIgG2b, and mIgG2c in 5 ng of purified mIgG were evaluated by ELISA. *n* = 5 per group. Data are means ± SD.

Figure S4. Shibuya et al.

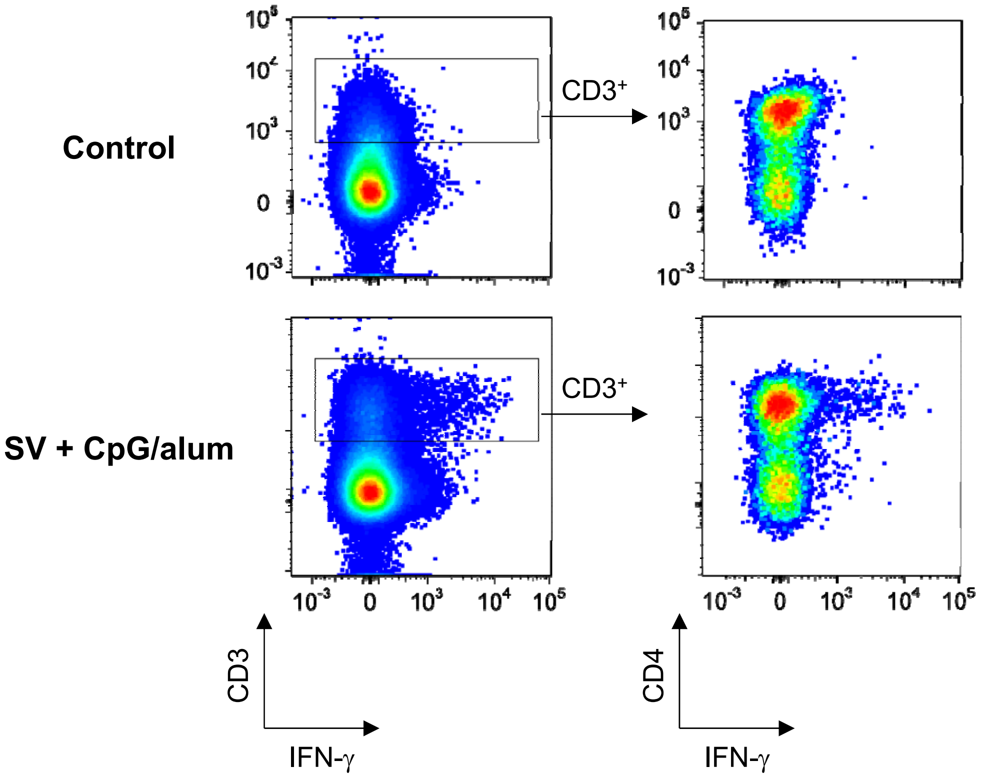

Figure S5. IFN-γ-producing cells in the lung, Related to Figure 4. After treatment with PBS or immunization with SV plus CpG/alum, mice were challenged with 1.2 × 10^3 $TCID_{50}$ PR8. Four days after the challenge, the mice were treated with Brefeldin A intraperitoneally followed by harvesting of the lung. Single cell suspensions samples were prepared and intracellular IFN-γ was analyzed in live $CD45^+$ cells by flow cytometry. Representative flow cytometry plots are shown.

Figure S5. Shibuya et al.

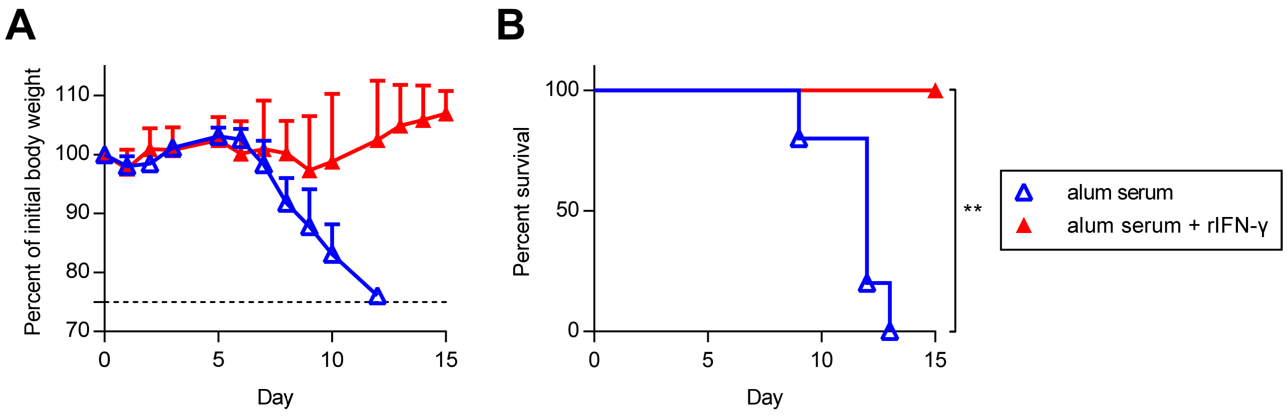

Figure S6. Requirement of IFN-γ for cross-protection, Related to Figure 4. A mixture of 1.2 × 10 $TCID_{50}$ PR8 and 2-fold diluted serum obtained from SV plus alum-immunized mice was administered to naïve mice intranasally. These mice were treated with recombinant IFN-γ, and we monitored **(A)** percentages of initial body weights and **(B)** survival for the next 15 days. **(A, B)** *n* = 5 per group. **(A)** Data are means ± SD. **(B)** ***P* <0.01 as indicated by comparing Kaplan-Meier curves using the log-rank test.

Figure S6. Shibuya et al.

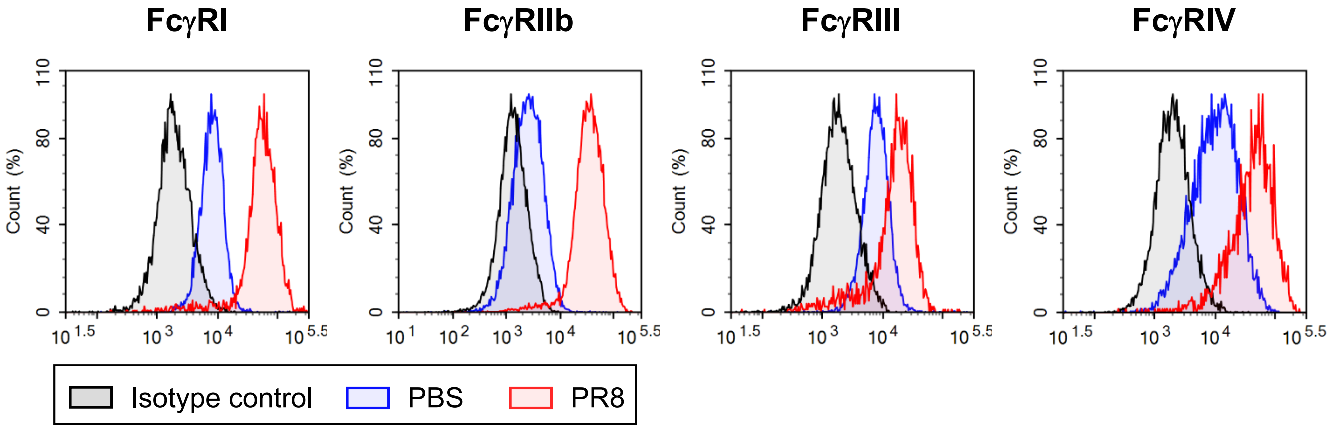

Figure S7. Expression change in FcγRs on alveolar macrophages after virus infection, Related to Figure 5. On day 5 after naïve mice were challenged with 1.2×10^3 $TCID_{50}$ PR8 or treated with PBS intranasally, the expression of FcγRI, FcγRIIb, FcγRIII, and FcγRIV on alveolar macrophages was determined by flow cytometry.

Figure S7. Shibuya et al.

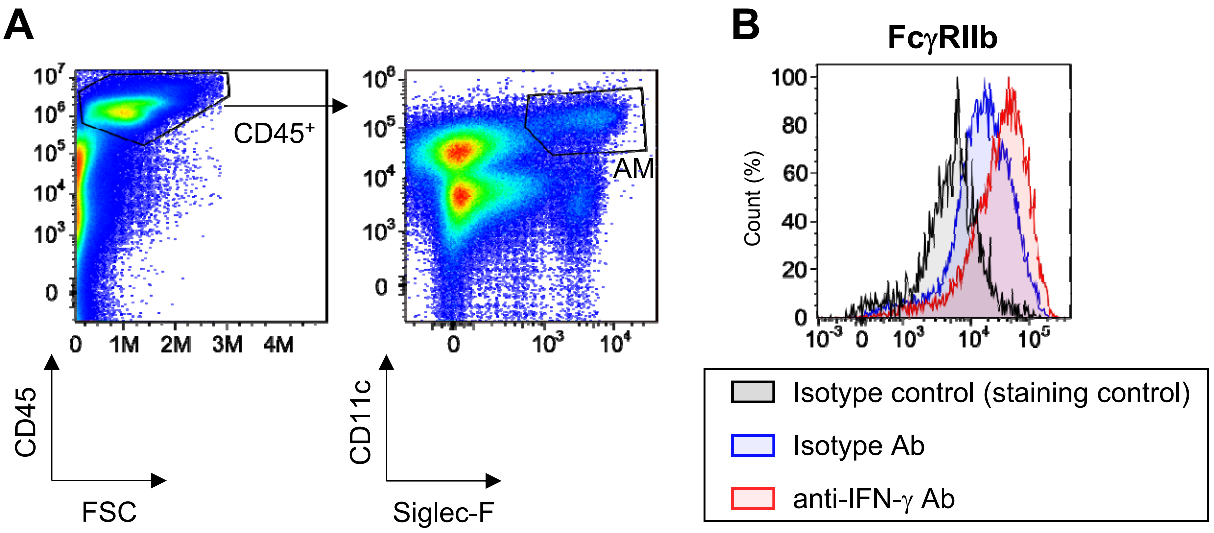

Figure S8. FcγRIIb expression on alveolar macrophages, Related to Figure 5. After immunization with SV plus CpG/alum, anti-IFN-γ antibody or isotype antibody was injected before PR8 challenge. Five days after PR8 challenge, the expression of FcγRs on alveolar macrophages were determined by flow cytometry. **(A)** Gating strategy for alveolar macrophages is shown. We defined alveolar macrophages in BALF as 7-AAD$^-$ CD45$^+$ CD11c$^+$ Siglec-F$^+$ cells. **(B)** Representative histogram of FcγRIIb staining on alveolar macrophages from data presented in Figure 5C is shown.

Figure S8. Shibuya et al.

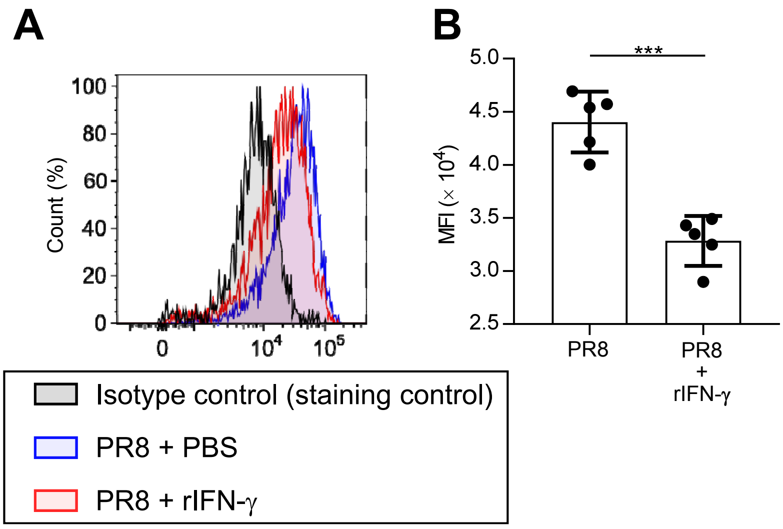

Figure S9. Expression change in FcγRIIb on alveolar macrophages after IFN-γ treatment, Related to Figure 5. Naïve mice were challenged with 1.2 × 10^3 $TCID_{50}$ PR8 and recombinant IFN-γ was administered intranasally on 4 day after the virus challenge. On day 5 after the challenge, the expression of FcγRIIb on alveolar macrophages was determined by flow cytometry. **(A)** Representative histogram of FcγRIIb staining on alveolar macrophages from data presented in Figure S9B and **(B)** FcγRIIb expression mean fluorescence intensity (MFI) is shown. **(B)** *n* = 5 per group. Data are means ± SD. ****P* <0.001 as indicated by Student's *t*-test.

Figure S9. Shibuya et al.
